# Genome-wide profiling of p53-regulated enhancer RNAs uncovers a subset of enhancers controlled by a lncRNA

**DOI:** 10.1038/ncomms7520

**Published:** 2015-03-27

**Authors:** Nicolas Léveillé, Carlos A. Melo, Koos Rooijers, Angel Díaz-Lagares, Sonia A. Melo, Gozde Korkmaz, Rui Lopes, Farhad Akbari Moqadam, Ana R. Maia, Patrick J. Wijchers, Geert Geeven, Monique L. den Boer, Raghu Kalluri, Wouter de Laat, Manel Esteller, Reuven Agami

**Affiliations:** 1 Division of Biological Stress Response, The Netherlands Cancer Institute, Plesmanlaan 121, 1066 CX Amsterdam, The Netherlands; 2 Doctoral Programme in Biomedicine and Experimental Biology, Centre for Neuroscience and Cell Biology, Coimbra University, 3004-504 Coimbra, Portugal; 3 Division of Cancer Epigenetics, Cancer Epigenetics and Biology Program (PEBC), Bellvitge Biomedical Research Institute (IDIBELL), L'Hospitalet de Llobregat, Barcelona, Catalonia 08908, Spain; 4 Department of Cancer Biology, University of Texas MD Anderson Cancer Center, Houston, Texas 77054, USA; 5 Department of Pediatric Oncology and Hematology, Erasmus University Medical Center, Erasmus MC, 3015 CE Rotterdam, The Netherlands; 6 Department of Cell Biology and Cancer Genomics Center, The Netherlands Cancer Institute, Plesmanlaan 121, 1066 CX Amsterdam, The Netherlands; 7 Hubrecht Institute-KNAW, University Medical Centre Utrecht, Uppsalalaan 8, 3584CT Utrecht, The Netherlands; 8 Department of Physiological Sciences II, School of Medicine, University of Barcelona, Barcelona, Catalonia 08036, Spain; 9 Institució Catalana de Recerca i Estudis Avançats (ICREA), Barcelona, Catalonia 08010, Spain; 10 Erasmus MC, Rotterdam University, 3000 CA Rotterdam, The Netherlands

## Abstract

p53 binds enhancers to regulate key target genes. Here, we globally mapped p53-regulated enhancers by looking at enhancer RNA (eRNA) production. Intriguingly, while many p53-induced enhancers contained p53-binding sites, most did not. As long non-coding RNAs (lncRNAs) are prominent regulators of chromatin dynamics, we hypothesized that p53-induced lncRNAs contribute to the activation of enhancers by p53. Among p53-induced lncRNAs, we identified LED and demonstrate that its suppression attenuates p53 function. Chromatin-binding and eRNA expression analyses show that LED associates with and activates strong enhancers. One prominent target of LED was located at an enhancer region within CDKN1A gene, a potent p53-responsive cell cycle inhibitor. LED knockdown reduces CDKN1A enhancer induction and activity, and cell cycle arrest following p53 activation. Finally, promoter-associated hypermethylation analysis shows silencing of LED in human tumours. Thus, our study identifies a new layer of complexity in the p53 pathway and suggests its dysregulation in cancer.

## Abstract (web summary)

Long non-coding RNAs (lncRNAs) have emerged as important regulators of gene expression through several distinct mechanisms. Here the authors further delineate the role of p53-induced lncRNAs within the p53-responsive pathways through the activation of enhancers.

## Graphical abstract

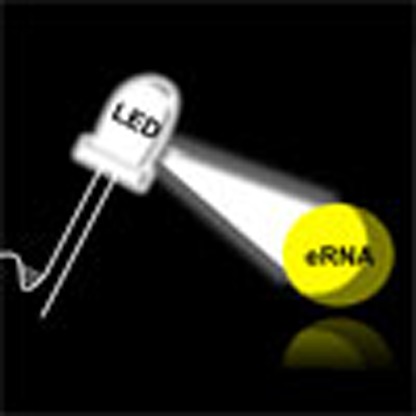

For several decades the foundations of molecular biology leaned against the dogma that genetic information is stored in protein-coding genes^1^. Although this concept was, and is still, largely true in prokaryotes, where genomes are mainly composed of protein-coding genes, it does not hold true for higher eukaryotes, where protein-coding sequences occupy less than 3% of the genome. Once considered transcriptionally inactive or simply referred to as ‘junk DNA’, the predominant fraction of the genome is in fact pervasively transcribed into thousands of different noncoding RNAs (ncRNAs), which can further be divided into two groups: small ncRNAs and long noncoding RNAs (lncRNAs). In addition, lncRNA genes have been classified based on the epigenetic state of their chromatin. For instance, the long intergenic noncoding RNAs (lincRNAs) are known for the presence of high histone 3 lysine 4 trimethylation (H3K4me3) at their promoters and high H3K36me3 along their transcribed regions, also referred as the K4K36 signature^2^. Alternatively, enhancer RNAs (eRNAs) are produced from transcriptionally active enhancer regions, which are epigenetically defined by high level of H3K4me1, low level of H3K4me3 (refs 3, 4) and high level of histone 3 lysine 27 acetylation (H3K27Ac) and H3K9Ac^5^.

Importantly, lincRNAs have recently emerged as potent regulators of gene expression. Recent publications have shown that lincRNAs are able to form complexes with various chromatin modifiers and to specifically direct them to different genomic regions. For example, the lincRNA-p21 was shown to interact with and guide the heterogeneous nuclear ribonucleoprotein K to repress a subset of p53 target genes^6^. However, although lincRNAs can mediate their effect in *cis* and in *trans*, eRNAs have been so far mainly characterized for their function in *cis*. Classically expressed as bidirectional transcripts from enhancer regions, eRNAs can alter the expression of their neighbouring genes through the formation of DNA loops, which help to bridge the interaction between enhancers and nearby promoters. Several transcription factors (TFs) were found to be important coordinators of eRNA expression^7^,^8^,^9^. An interesting case revealed that the tumour-suppressor p53 directly regulates the expression of eRNAs upon cellular stresses^9^.

*P53* function is frequently compromised in tumours, in part as a consequence of somatic mutations, which occur in more than 50% of all human cancers^10^. Moreover, it was also shown that p53 is inactivated in various cancers by dysregulation of its regulatory pathway, such as the amplification and over-expression of its negative regulators MDM2 and MDM4 (refs 11, 12). Upon cellular stresses, p53 is activated and acts primarily as a TF to mediate and coordinate a complex transcriptional response that regulates hundreds of target genes. Until recently, the p53 network was mainly characterized by its impact on protein-coding target genes^13^. However, we now begin to discover and appreciate the great potential of ncRNAs in the intricate regulatory network of p53. The recent discoveries that p53 can mediate its function in collaboration with diverse lncRNAs, suggest a potential role for this novel regulatory layer in disease such as cancer, and therefore urge the importance of an in-depth reassessment of the p53 transcriptional response.

Here, by using Global Run-On sequencing (GRO-seq), we mapped p53-responsive enhancers bound by p53. Surprisingly, we also found a large group of p53-activated enhancers that were not associated with p53. Although motif-search analysis identified the p53 signature in the enhancers bound by p53, no single TF was found to govern the majority of p53-unbound enhancer groups. However, further analysis revealed that nutlin-3a-induced Signal transducer and activator of transcription 3 (STAT3), B-cell lymphoma 3-encoded protein (BCL3), FBJ murine osteosarcoma viral oncogene homolog (FOS) might largely contribute to the transcriptional regulation of indirect p53 target genes. Next, we assessed whether p53-responsive lncRNAs could play a role in p53-mediated enhancer activation. Our data revealed that a prominent p53-induced lncRNA termed LED (LncRNA activator of Enhancer Domains) is required for p53-induced cell cycle arrest and is involved in the activation of a subset of p53-bound and unbound enhancers by inducing an epigenetic change. Strikingly, promoter-associated hypermethylation of LED was uncovered in several cancer cell lines and human tumours with preference to p53 wild-type (WT) status, suggesting its implication in tumorigenesis. Altogether, we propose that LED is an important regulator and a potential tumour suppressor of the p53 pathway.

## Results

### Genome-wide identification of p53-regulated eRNAs

**Figure 1:**
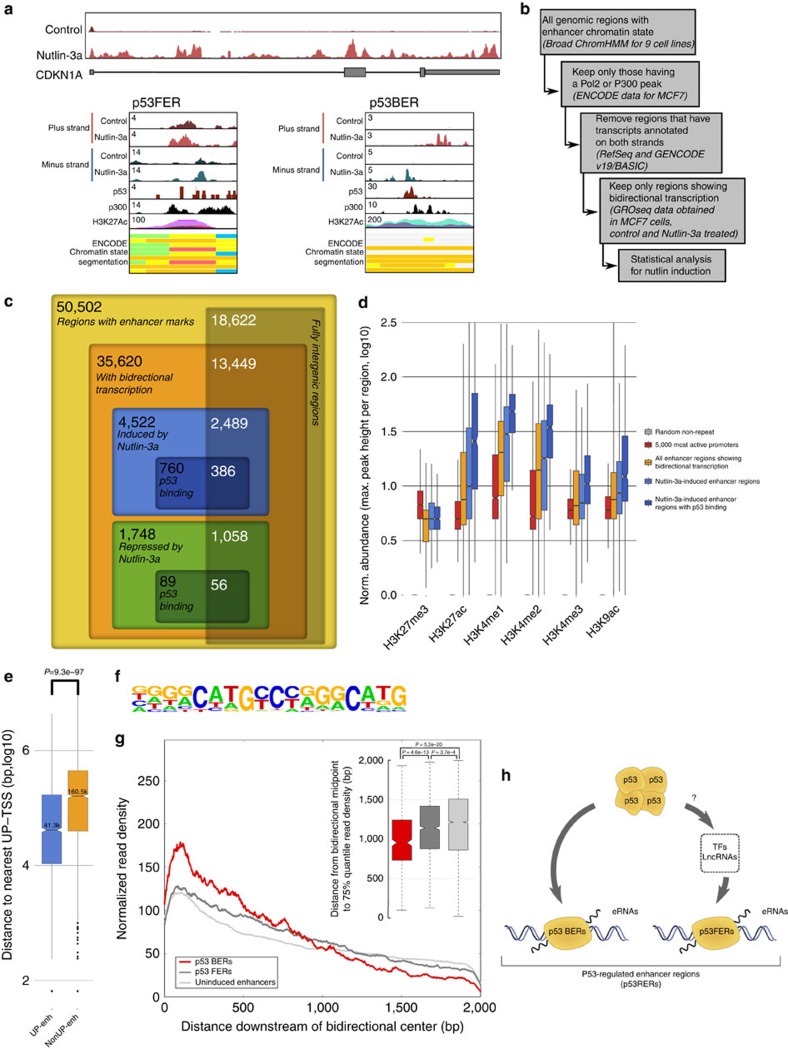
Identification of p53-regulated enhancer RNAs (p53RERs). (**a**) GRO-Seq snapshot showing the induction of CDKN1A/p21 transcription upon 12 h nutlin-3a treatment (upper scheme). Display of nutlin-3a-induced bidirectional transcription at p53-bound and unbound enhancers (lower schemes). Binding of p300, presence of H3K27 acetylation and chromatin states in nine cell lines are also presented. (**b**) Diagram showing the outline of the algorithm to identify enhancers using chromatin segmentation data, and ChIP-Seq and GRO-Seq data. (**c**) Venn diagram showing the number of retrieved regions at the steps of the enhancer identification algorithm. (**d**) Boxplot showing the abundance of several enhancer marks at different regions (grey: 5,000 random non-repeat regions; red: promoters of the 5,000 most abundant genes as identified by GRO-seq in the nutlin-3a-treated condition; orange: all putative enhancer regions showing bidirectional transcription; blue: subset of the putative enhancers showing significant induction upon nutlin-3a treatment (induced p53RERs); dark blue: subset of the induced p53RERs having a p53 peak within 1 kb (p53BERs). (**e**) Boxplot showing the distances between enhancer region and the nearest annotated gene induced upon nutlin-3a treatment, for induced p53RERs (UP Enhancers) and nutlin-3a unresponsive enhancers (nonUP enhancers). (**f**) Motif identified in induced p53BERs using HOMER. (**g**) Normalized density of transcription downstream of the point of bidirectional transcription for p53BERs (red), p53FERs (dark grey) and uninduced enhancers (light grey). The lines indicate the median across all regions. The boxplot in the inset shows the distance from the point of bidirectional transcription to the 75% quantile of read density. (**h**) Schematic representation of p53-regulated enhancers (p53RERs). P53 can directly bind to enhancers (p53BERs) or regulated intermediate factors (for example, lncRNAs or TFs) to indirectly influence another subset of enhancers (p53FERs).

To detect active enhancers, we relied on the observation that eRNA production marks enhancer activity. Using GRO-seq of MCF-7 cells treated with nutlin-3a, a specific activator of p53, we obtained a genome-wide quantitative snapshot of transcriptional activity. As expected, the activation of CDKN1A/p21, and many other known target genes of p53, was readily apparent (Fig. 1a). Moreover, also several previously described p53-induced eRNAs could be confirmed (Fig. 1a)^9^. We proceeded to generate a global view of the putative p53-regulated enhancers in MCF-7 cells. By taking the union of the enhancer domains defined by the Broad chromatin segmentation^14^, we selected only regions showing RNA polymerase II (RNAPII) and p300 binding in MCF-7 cells (using publicly available chromatin immunoprecipitation sequencing (ChIP-seq) data^15^) and excluded those having annotated transcripts on both strands, as well as those having transcription start sites. The remaining regions were extended by 1 kb for the purpose of read counting and used in conjunction with the aforementioned GRO-seq data (Fig. 1b). This analysis resulted in the detection of 50,502 putative enhancers of which 6,270 were regulated (at least one direction) by nutlin-3a treatment, and referred here as p53-regulated enhancer regions or p53RERs.

Since p53 mainly functions as an activator of transcription^16^, the vast majority (72%) of the differentially expressed eRNAs showed induction upon nutlin-3a treatment, and activated enhancers were more often bound by p53 than the repressed enhancers (Fig. 1c). Using ENCODE ChIP-seq data obtained from MCF-7 cells, the presence of enhancer-specific histone modifications was confirmed (Fig. 1d). Moreover, on average, nutlin-3a-induced enhancers are positioned closer to p53-regulated canonical genes (median 41 kb), than to non-regulated genes (median 161 kb; Fig. 1e). This observation is in agreement with the notion that eRNAs are potent regulators of neighbouring target genes^7^,^8^,^9^. In support of a role for nutlin-3a-regulated enhancer regions within the p53 pathway, the gene ontology analysis on neighbouring genes revealed enrichment for genes involved in DNA damage response/signal transduction by p53 (GO term 0030330, *P*=1.6e−3). Moreover, a *de novo* motif analysis using HOMER^17^ confirmed the presence of a p53 response element at p53-bound enhancer regions (p53BERs; Fig. 1f).

Next we reanalysed published p53 ChIP-seq data^15^ to identify which of the p53RERs were direct targets of p53 (hereafter referred to as p53BERs). The enhancers in the remaining subset of p53RERs were considered p53-free enhancer regions, or p53FERs, as no enrichment for p53 or any known TF signature was found. Intriguingly, activation of p53BERs and p53FERs was different, as we observed a faster transcription drop-off for the first group compared with the second (Fig. 1g and Supplementary Fig. 1a). Although we did not further investigate this difference, we suggest that it reflects a secondary (p53-indirect) and, thus, differentially regulated wave of transcription (Supplementary Fig. 1b). Therefore, we hypothesized that activation of p53FERs could be mediated by a combination of several different factors (Fig. 1h). Indeed, the analysis of TF-binding sites (using the ENCODE Uniform TFBS data) showed that while several TFBSs were enriched in induced p53RERs, with respect to all enhancers, no TFBS was specifically enriched in the p53-free enhancer group (Supplementary Fig. 1c). However, further analysis revealed that among these potential regulators, three (STAT3, BCL3 and FOS) were regulated by nutlin-3a (Supplementary Fig. 1d,e). Intersection between their binding sites and p53FERs revealed that STAT3, BCL3 and FOS may directly regulate 55% of these enhancer regions (Supplementary Fig. 1f). Interestingly, despite their significant contribution, the transcriptional regulation of a large number of p53FERs remains elusive. An additional or complementary possibility is that the transcriptional activation of enhancers is mediated by p53-dependent lncRNAs, as lncRNAs were recently shown to be able to associate with and modulate regulatory elements^18^,^19^.

### LED is required for the p53 transcriptional response

**Figure 2:**
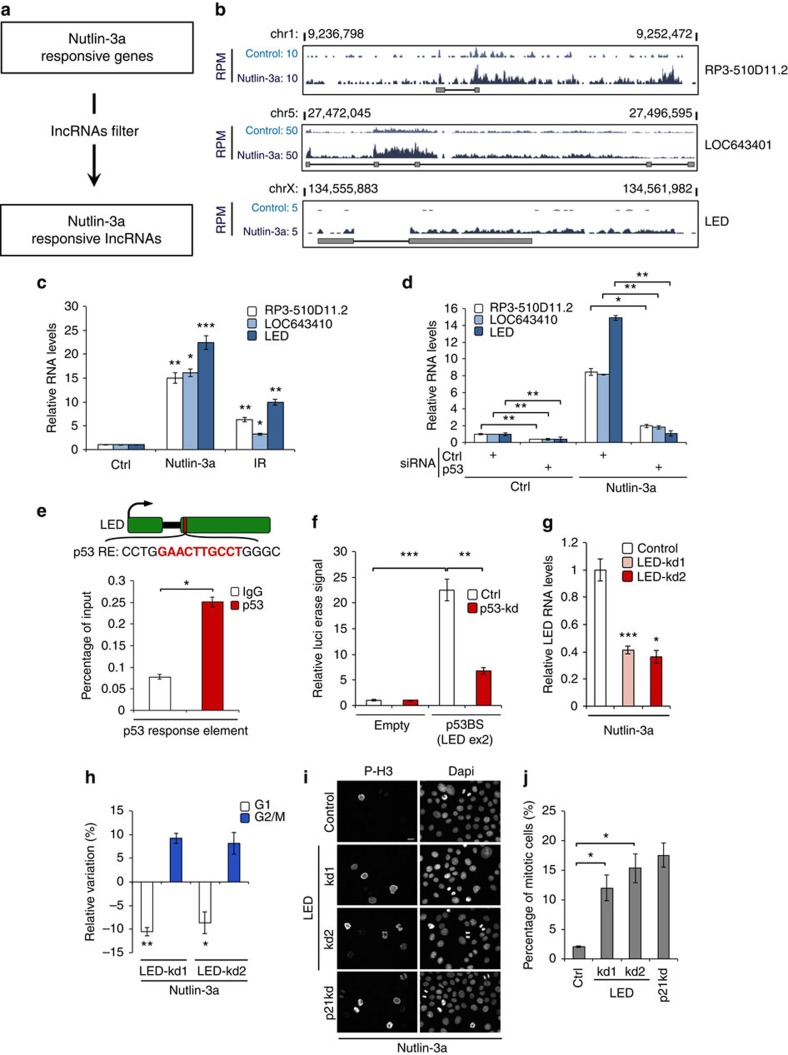
Novel p53-regulated lncRNA LED. (**a**) Outline of pipeline for identification of p53-regulated lncRNAs. MCF-7 cells were treated with 8 μM nutlin-3a for 12 h and subjected to RNA sequencing (RNA-seq). (**b**) Display of genomic location and RNA-seq data showing the nutlin-3a induction of selected lncRNAs in MCF-7 cells. Values are represented by RPM (reads per million). (**c**) Stress-dependent regulation of selected lncRNAs upon nutlin-3a (8 μM) and ionizing radiation (IR; 10 Gy) treatment in MCF-7 cells measured by quantitative reverse transcription–PCR (qRT–PCR). Values are represented by fold induction (*n*=3; ***P<0.005, **P<0.01, *P<0.05, two-tailed Student’s *t*-test). (**d**) P53-dependent regulation of validated lncRNAs in MCF-7 cells transfected with a control (Ctrl) or p53 siRNA in the presence or absence of nutlin-3a. Values are represented by fold induction (*n*=3; **P<0.01, *P<0.05, two-tailed Student’s *t*-test). (**e**) Schematic representation of p53 response element (p53 RE) in LED gene body. Chromatin immunoprecipitation performed in nutlin-3a-treated MCF-7 cells using IgG or p53 antibodies followed by qPCR in the p53 RE region. Values represent the percentage of input (*n*=3; *P<0.05, two-tailed Student’s *t*-test). (**f**) MCF-7 cells were co-transfected with an empty or LED (exon 2) p53BS pGL3-basic vector and either a Ctrl or p53-targeting siRNA. The relative luciferase activities (Firefly/Renilla) were normalized to the Ctrl reaction (empty pGL3-basic vector; *n*=3; ***P<0.005, **P<0.01, two-tailed Student’s *t*-test). (**g**) qRT–PCR measuring relative LED RNA levels in MCF-7 cells transfected with a Ctrl or two independent LED siRNAs (LED-kd; *n*=3; ***P<0.005, *P<0.05, two-tailed Student’s *t*-test). (**h**) Relative cell cycle variation (LED-kd minus control-kd) of MCF-7 cells transfected with a Ctrl or two independent LED siRNAs, treated with nutlin-3a for 12 h. To capture cycling cells in G2/M, cells were treated with nocodazole for 24 h, before flow cytometric analysis (*n*=3; **P<0.01, *P<0.05, two-tailed Student’s *t*-test). (**i**) Immunostaining detection of the mitotic marker phospho-histone H3 ser10 (P-H3) and 4,6-diamidino-2-phenylindole (DAPI) staining in MCF-7 cells treated as in **h** and using a siRNA targeting p21 (p21kd) as a positive Ctrl. Scale bar, 25 μM. (**j**) Quantification of the marker P-H3 from **i** indicating the percentage of mitotic cells (*n*=3; *P<0.05, two-tailed Student’s *t*-test).

We therefore set to identify relevant lncRNAs by profiling the transcriptome of nutlin-3a-treated MCF-7 cells. Using RNA-sequencing (RNA-seq) in combination with an annotation catalogue comprised of Ensembl, Refseq and the Broad Linc Catalog^20^, we identified 194 nutlin-3a-responsive lncRNA genes (Fig. 2a and Supplementary data 1). We then reasoned that the most upregulated transcripts might have a greater biological importance, and consequently selected the top three most activated lncRNAs (that is, RP3-510D11.2, loc643401 and linc00086 (hereinafter referred to as LED)) for further characterization (Fig. 2b). Interestingly, these three lncRNAs were also recently identified, but not functionally assessed, in two genome-wide studies performed in HCT-116 and Cal-51 cancer cell lines^21^,^22^. Validation confirmed that the selected lncRNAs were induced in MCF-7 cells upon both nutlin-3a and ionizing radiation treatment (Fig. 2c). Similar results were also obtained in ZR-75-1 and MALM-3M cell lines (Supplementary Fig. 2a). Next, we determined whether these lncRNAs were regulated by p53. As expected, we observed that p53 depletion decreased both the basal and nutlin-3a-induced levels of all tested lncRNAs (Fig. 2d). Moreover, we demonstrated the direct binding of p53 at each lncRNA locus by using publicly available p53 ChIP-seq data and ChIP-quantitative PCR (qPCR; Fig. 2e and Supplementary Fig. 2b). Using a luciferase reporter, we also showed the p53-dependent promoter activity of the p53 response element found in LED exon 2 (Fig. 2f). Altogether, these results demonstrate that our selected lncRNAs are *bona fide* p53 targets.

Next, we assessed whether the depletion of our selected p53-induced lncRNAs phenotypically influenced the p53 transcriptional response using short interfering RNAs (siRNAs). Among the investigated candidates, only LED significantly influenced the G1 checkpoint arrest following nutlin-3a treatment, as shown by flow cytometry (Fig. 2g,h). To corroborate this finding, we first evaluated cellular entry into mitosis using phospho-H3 (ser10) staining. Cells treated with siRNAs targeting LED showed a significant increase of phospho-H3 (ser10) compared with cells transfected with a non-targeting siRNA (Fig. 2i,j). Furthermore, cell proliferation assays confirmed this observation, as LED-suppressed cells proliferated more following nutlin-3a treatment in comparison with control-transfected cells (Supplementary Fig. 2c,d). Thus, the induction of LED lncRNA is required for efficient sustenance of p53 transcriptional response.

**Figure 3:**
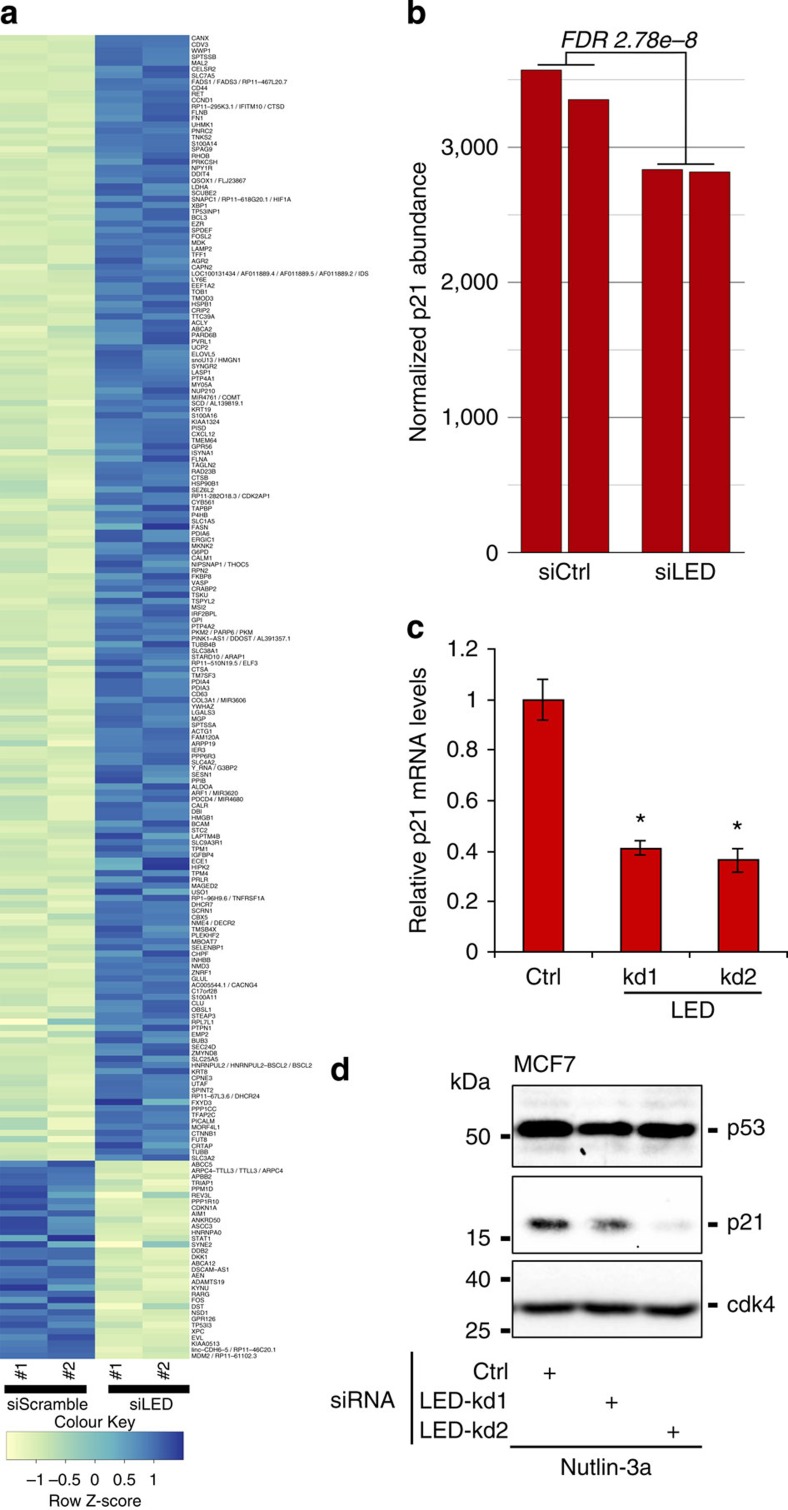
LED is required for the proper p53 transcriptional response. (**a**) RNA sequencing heatmap showing a subset of genes differentially expressed upon LED knockdown in MCF-7 cells treated 12 h with nutlin-3a (only the subset with absolute fold-change>30% and FDR<5% is shown). (**b**) Barplot derived from the RNA-sequencing showing the normalized p21 mRNA levels in control and LED knockdown conditions. (**c**) Relative mRNA levels of p21 upon transfection of a control or two independent LED siRNAs (LED-kd), measured by qRT–PCR in MCF-7 cells treated with Nutlin-3a for 12h (*n*=3; *P<0.05, two-tailed Student’s *t*-test). (**d**) Western blot showing p53 and p21 protein levels in MCF-7 cells transfected with a control or two independent LED siRNAs (LED-kd) and treated with nutlin-3a for 12 h. Detection of CDK4 protein levels is also displayed.

To investigate the mechanism by which LED impacts the p53 transcriptional response, we performed gene expression analysis by RNA-seq following knockdown of LED in MCF-7 cells treated 12 h with nutlin-3a. A total of 1,983 genes were responsive to LED depletion (FDR less than 1%), of which 1,340 were upregulated and 643 downregulated (Fig. 3a). Interestingly, LED knockdowns significantly reduced the levels of the cell-cycle regulator p21 (FDR of 2.7e-8; Fig. 3b), without influencing p53 levels (FDR of 0.12). We further validated this observation by showing the LED-dependent regulation of p21 at both the mRNA (Fig. 3c) and protein levels (Fig. 3d). Similar results were also obtained in ZR-75-1 and MALM-3M cell lines (Supplementary Fig. 3). Altogether, our results indicate that LED is required for an efficient p53-dependent checkpoint by maintaining high levels of p21.

### LED associates with and regulates enhancer domains

**Figure 4:**
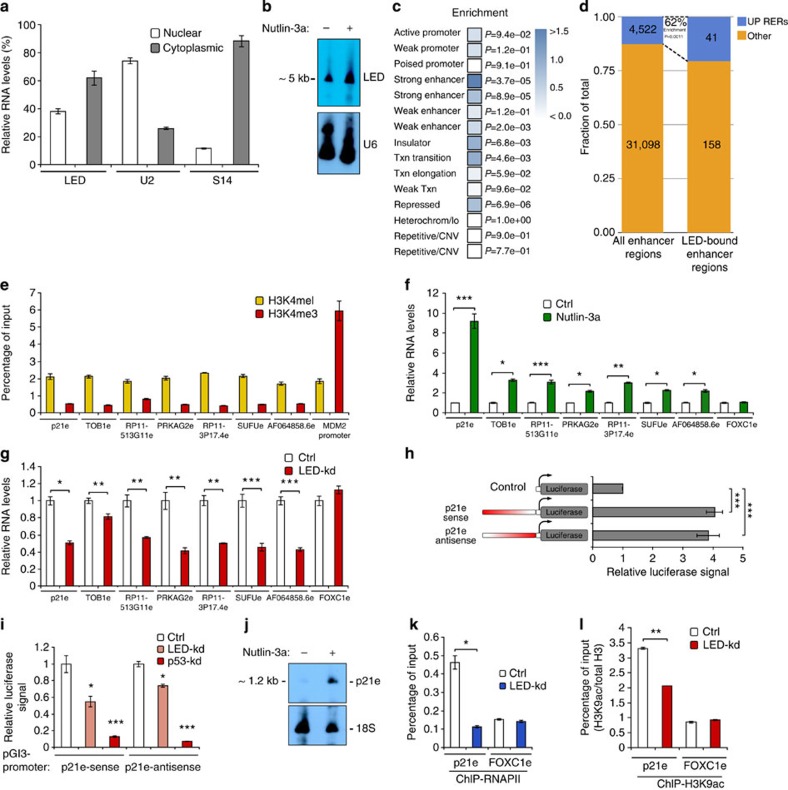
LED binds preferentially to enhancers and regulates enhancer RNA production from p53RERs. (**a**) LED subcellular localization in MCF-7 cells treated with nutlin-3a. U2 and S14 genes were used as controls (Ctrl) for nuclear and cytoplasmic fraction, respectively. (**b**) Northern blot analysis showing LED transcript in MCF-7 cells incubated or not with nutlin-3a. U6 was used as a loading Ctrl. (**c**) Enrichment of LED ChIRP peaks in genomic features defined by ENCODE. (**d**) Bar graph showing the fraction of induced p53RERs of all found putative enhancer regions (left) and of all LED-bound enhancer regions (right). The enrichment of induced p53RERs in the LED-bound fraction is significant with *P*=0.0011 (hypergeometric distribution). (**e**) Quantification of H3K4me1 and H3K4me3 at LED-associated p53RERs by ChIP-qPCR in MCF-7 cells. Values were corrected to total H3 and MDM2 promoter was used as a Ctrl. Mean±s.d. are shown. (**f**) Nutlin-3a regulation of LED-associated p53RERs expression in MCF-7 cells. FOXC1e was used as a negative Ctrl (*n*=3; ***P<0.005, **P<0.01, *P<0.05, two-tailed Student’s *t*-test). (**g**) LED-dependent regulation of p53RERs upon nutlin-3a treatment in MCF-7 cells transfected with a Ctrl or LED siRNA. FOXC1e was used as a negative Ctrl (*n*=3; ***P<0.005, **P<0.01, *P<0.05, two-tailed Student’s *t*-test). (**h**) MCF-7 cells were transfected with an empty, p21e-sense or p21e-antisense reporter construct. The firefly/renilla luciferase activities were normalized to the Ctrl reaction (*n*=3; ***P<0.005, two-tailed Student’s *t*-test). (**i**) MCF-7 cells were co-transfected with an empty, p21e-sense or p21e-antisense pGL3-promoter vector and either a Ctrl, LED siRNA or p53 siRNA. The relative luciferase activities were normalized to the Ctrl reaction (empty vector) and subsequently to the Ctrl siRNA (*n*=3; ***P<0.005, *P<0.05, two-tailed Student’s *t*-test). (**j**) Northern blot analysis showing p21e antisense transcript in MCF-7 cells treated or not with nutlin-3a. 18S was used as a loading Ctrl. (**k**) Quantification of RNAPII binding at p21e and FOXC1e regions by ChIP-qPCR. MCF-7 cells were transfected with a Ctrl or LED siRNA and treated with nutlin-3a (*n*=3; *P<0.05, two-tailed Student’s *t*-test). (**l**) Quantification of H3K9Ac at p21e and FOXC1e regions by ChIP-qPCR. MCF-7 cells were transfected with a Ctrl or LED siRNA and treated with nutlin-3a. Values were normalized to total H3 (*n*=3; **P<0.01, two-tailed Student’s *t*-test).

Next, to investigate whether LED, a *bona fide* lncRNA of ~5 kb, exerts its function in the nucleus or in the cytoplasm, we examined its subcellular localization (Fig. 4a,b and Supplementary Fig. 4a,b). As LED is partially located in the nucleus, we set out to assess its putative interaction with chromatin. We performed chromatin isolation by RNA purification technique (ChIRP)^23^ using anti-sense oligos to LED (odd and even) or the bacterial β-galactosidase (lacZ) and confirmed the specific enrichment for LED, but not glyceraldehyde 3-phosphate dehydrogenase RNA (Supplementary Fig. 4c). Then, we sequenced the DNA fragments co-purified in the two pools, aligned reads to the genome and processed them using peak calling software in a pipeline developed for ChIRP-seq data. Overlap of the peaks from the odd and even purifications indicated LED binding in 1,698 putative sites (Supplementary data 2). To investigate the nature of the genomic features present at LED-associated domains, we made use of chromatin state annotations previously defined by Ernst and colleagues^14^. Intriguingly, although LED-associated sites were present in all chromatin states, significant enrichment was observed in strong enhancer regions (Fig. 4c and Supplementary Fig. 4d). Moreover, intersection with the GRO-Seq data revealed that a subgroup of LED-bound enhancers was sensitive to nutlin-3a (Fig. 4d). Interestingly, further analysis showed that this subgroup was partially overlapping with p53, STAT3, BCL3 and FOS (Supplementary Fig. 4e). This observation suggests LED as a co-factor in the nutlin-3a-dependent regulation of enhancers.

To assess the regulatory potential of LED on enhancers, we first selected a subset of LED-associated enhancer domains (Supplementary Fig. 4f). Then, we reasserted that bound enhancers harbour hallmarks of active enhancers^3^,^24^,^25^ (Supplementary Fig. 4g). Furthermore, we performed ChIP for H3K4me1 and H3K4me3, to confirm the relative deposition of these histone modifications in our cell system (Fig. 4e). Next, we tested a selected group of LED-associated enhancers for eRNA production by quantitative reverse transcription–PCR; after DNase-treatment of RNA isolated from MCF-7 cells incubated with or without nutlin-3a. As previously observed with the GRO-Seq, this analysis confirmed the nutlin-3a-dependent transcriptional induction of eRNAs at all tested LED-associated enhancers (Fig. 4f). This nutlin-3a induction of eRNAs was specific, as the abundance of a control, LED-unbound, FOXC1 enhancer (FOXC1e) RNAs remained unaffected. Strikingly, RNA interference-mediated LED knockdown reduced the level of activation of these putative eRNAs (Fig. 4g), indicating direct regulation of eRNA production by LED. Intriguingly, we noticed among the LED-associated enhancers a prominent peak located within the first intron of p21. We further validated the association of LED to p21 enhancer (p21e) domain using ChIRP-qPCR (Supplementary Fig. 4 h). To evaluate the enhancing potential of p21e, we cloned a 1.2-kb fragment into a pGL3-promoter luciferase reporter vector. As expected from an enhancer domain, p21e activated the luciferase gene in an orientation-independent manner (Fig. 4h). A detailed analysis of p21e 1.2 kb fragment revealed the presence of a p53 response element overlapping LED-binding site (Supplementary Fig. 4i). Thus, we suggested that both LED and p53 may participate in regulating p21e enhancing activity. Indeed, we demonstrated that LED or p53 knockdown decreases the enhancing activity of both sense and antisense p21e luciferase reporters (Fig. 4i). Using northern blotting and RNAPII ChIP experiments, we further supported the presence of an antisense eRNA at p21e locus and its regulation by nutlin-3a and LED (Fig. 4j,k and Supplementary Fig. 4j). Last, we examined whether p21e could interact with distant promoters by DNA looping, using circular chromosome conformation capture (4C) experiments. This analysis failed to reveal long-distance enhancer–promoter interactions, suggesting that p21e acts within its functional domain on the p21 promoter (Supplementary Fig. 4k). Collectively, these results demonstrate that LED associates with chromatin regions marked as enhancers and regulates the production of eRNAs.

To further delineate the mode of action by which LED regulates enhancers, we hypothesized that LED controls enhancer activity by remodelling the epigenetic state of enhancer domains. To investigate this possibility, we assessed whether LED influences the deposition of active enhancer histone marks, such as H3K27ac and H3K9ac. ChIP analyses revealed that the levels of H3K9ac, but not H3K27ac, were decreased at p21 enhancer domain upon LED knockdown (Fig. 4l and Supplementary Fig. 4l). Similar results were also obtained with another LED-associated enhancer (Supplementary Fig. 4m). Interestingly, in concomitance with H3K9ac reduction, we also noticed a lower p53-binding affinity at distal and proximal enhancers located upstream of p21 transcription start site (Supplementary Fig. 4n,o). These results indicate that LED may influence the production of eRNAs, by influencing the deposition of H3K9ac at specific enhancer loci. In addition or as a consequence of its influence on H3K9ac, LED may influence the binding of TFs at or in the vicinity of enhancer domains.

### LED is inactivated by promoter hypermethylation in cancer

**Figure 5:**
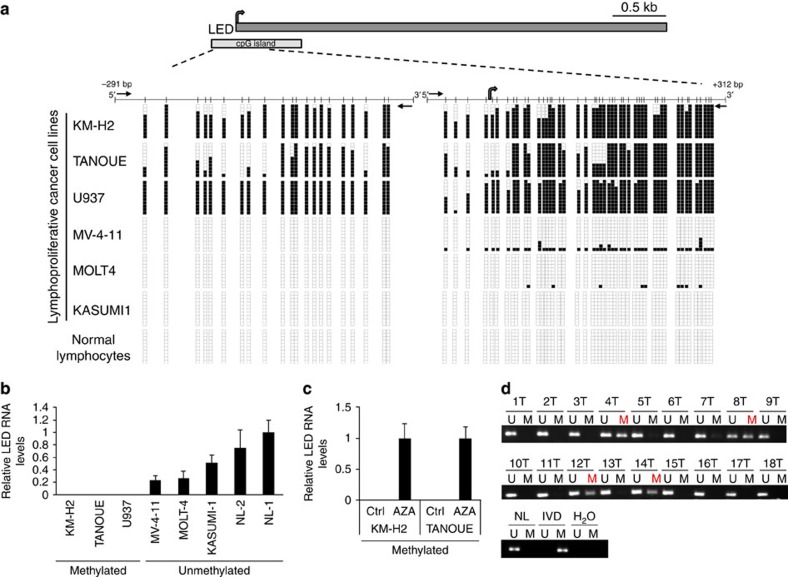
DNA methylation-associated silencing of LED in lymphoproliferative tumours. (**a**) Schematic representation of LED genomic loci and CpG island. Bisulfite genomic sequencing analysis of LED CpG island in human lymphoproliferative cancer cell lines and normal lymphocytes as tissue control (Ctrl). Location of bisulfite genomic sequencing PCR primers (black arrows), CpG dinucleotides (vertical lines) and the transcriptional start site (grey arrow) are shown. Ten single clones are represented for each sample. Presence of unmethylated or methylated CpGs is indicated by white or black squares, respectively. (**b**) LED expression levels in methylated or unmethylated human lymphoproliferative cell lines and in normal lymphocytes (NLs) as Ctrl. Values were determined by quantitative reverse transcription–PCR (qRT–PCR) in triplicates and are expressed as mean±s.e.m. (*n*=2–4). (**c**) Restored LED expression after treatment with DNA demethylating agent 5-aza-2′-deoxycytidine (AZA) in LED CpG island methylated cell lines. Values were determined in triplicates by qRT–PCR and are expressed as mean±s.e.m (*n*=3). (**d**) Methylation-specific PCR analyses for LED methylation in primary leukaemias. The presence of a band under the U lane indicates unmethylated alleles, whereas the presence of a band under the M lane indicates methylated alleles. Normal lymphocytes and *in vitro* methylated DNA (IVD) are shown as negative and positive Ctrls for methylated alleles, respectively.

Gene expression comparison analysis suggests not only that LED is activated by p53, but also that its function is intimately linked to the transcriptional response of p53. We therefore examined whether LED is inactivated in cancer. Inspection of the LED promoter sequence identified a large CpG island region (Fig. 5a). As CpG islands are often subject to hypermethylation and silencing, we asked whether LED promoter hypermethylation leads to a reduced LED expression in cancers. We first measured the methylation status of LED CpG islands in 135 cancer cell lines covering a wide range of cancers. Notably, we find LED promoter methylation in ~44% (59/135) of all tested cell lines, with a large proportion in leukaemia (Supplementary Fig. 5a–c). Moreover, we observed a strong preference for methylation in p53 WT cell lines (60%; 29/48) as compared with p53 mutants (34%; 30/87, *P*=0.004 (χ^2^); Fig. 5a, Table 1). Most importantly, we then assessed and validated the transcriptional silencing of LED by its promoter-associated hypermethylation on several cancer cell lines. As expected, there was a significant anti-correlation between LED expression and its methylation status (Fig. 5b and Supplementary Fig. 5d,e). Also, treatment of LED-promoter-hypermethylated cell lines with the DNA-demethylating agent 5-Azacytidine resulted in LED re-expression (Fig. 5c). Moreover, we observed that methylation-dependent inactivation of LED may delay or reduce the induction of p21 mRNA, as compared with unmethylated cell lines (Supplementary Fig. 5f). Finally, we evaluated the prevalence of LED-promoter hypermethylation in various human tumours. Using methylation-specific PCR, we observed LED-promoter hypermethylation in various tumour types, most prominently reaching 22% of all samples in acute lymphocytic leukaemia (ALL; Fig. 5d and Table 2).

## Discussion

Coordination of gene expression components within response programmes is a delicate task crucial for the maintenance of cellular homeostasis. One key player for such coordination is the tumour-suppressor p53, which organizes the implementation of an appropriate cellular response to stress cues such as DNA damage and emerging oncogenes. With the discovery that the genome is pervasively transcribed^26^, it is likely that novel p53-sensitive transcripts and regulatory networks will be uncovered. Although many target promoters of p53 are well-established, little is known about the role of this master tumour suppressor as enhancer factor.

ERNAs were recently suggested as transcriptional regulators^9^,^27^. Moreover, eRNA level emerges as robust readout for determining enhancer activity, as it correlates with the expression levels of neighbouring target genes. The GRO-seq is a very powerful technique that can be used to globally measure newly synthesized eRNAs and to infer enhancer activity in a genome-wide manner. Here we used GRO-seq to map and quantify eRNAs induced by the p53 inducer nutlin-3a, and identified hundreds of regulated enhancer domains. Although many enhancers are direct targets of p53, most nutlin-3a-regulated enhancer domains were not bound by this TF. Thus, it is likely that those enhancers are bound and influenced by factors regulated by p53. In this respect, bioinformatics analyses revealed three TFs (STAT3, BCL3 and FOS) with potential regulatory impact on p53-free enhancer regions. However, despite the potential combined influence of these three TFs on approximately 55% of p53FERs, the regulation of a large fraction remains unexplained. In search of novel p53FER regulators, we discovered LED, a lncRNA induced by p53, and subsequently demonstrated its involvement in the regulation of p53-sensitive enhancers, including both p53BERs and p53FERs. In support of our finding, two recent studies reported that not only TFs but also *trans*-acting lncRNAs are present at transcriptional regulatory regions^18^,^19^. For instance, the lncRNA Paupar was found to interact with the TF PAX6 at enhancer domains in order to modulate the expression of genes involved in neural stem cell fate.

Thus, for the first time, we demonstrate the contribution of a p53-induced lncRNA, termed LED, in the regulation of enhancer-derived transcripts.

LED is a direct transcriptional target of p53. Suppression of LED expression attenuated the activation of target enhancer domains, as demonstrated by reduced eRNA production and by a lower H3K9 acetylation. We found that LED was associated with different genomic loci and especially enriched at enhancer domains producing eRNAs. Notably, a subgroup of these enhancers is regulated by p53. Moreover, some, but not all, LED-bound enhancers were concomitantly bound by p53. Despite this observation, all tested LED-bound p53-induced eRNAs responded to siRNA-mediated LED depletion. This suggests that LED is a p53-induced factor that contributes to both the direct and indirect p53 transcriptional response.

How exactly does LED trigger enhancer activation? Modulation of the chromatin epigenetic state plays an important role in the regulation of gene expression. Thus far, several studies have put forward the idea that lncRNAs are important epigenetic regulators. For example, the lncRNA HOTAIR represses gene expression by interacting with and guiding the polycomb repressive complex 2 to target promoters, where it contributes to chromatin compaction by catalysing the methylation of histone H3 at lysine 27 (ref. 28). Alternatively, ribonucleoprotein complexes such as HOTTIP:MLL/WDR5 activate gene expression by promoting the deposition of an active mark (H3K4me3) on promoters^29^. Here we complement these observations by showing that LED is essential for the acetylation of H3K9 at bound enhancers, a modification associated with active gene transcription. Moreover, the p21 locus analysis also revealed the potential implication of LED in the epigenetic regulation of nearby contacted enhancers. This finding is consistent with the fact that LED is required for proper p53 binding at p21 upstream enhancers, as well as for RNAPII loading and eRNA transcription at bound enhancers. Moreover, genome-wide deposition of H3K9 acetylation was previously reported to be enriched at regulatory elements such as promoters, enhancers and repetitive sequences^14^. Consequently, active transcriptional programmes may primarily be epigenetically governed by the action of a subset of activating lncRNAs. However, whether LED influences the epigenetic features of regulatory elements before the TF-binding dysregulation, remains to be elucidated.

P53 is one of the most commonly inactivated gene in human cancer, with somatic mutations occurring in approximately half of all human cancers^30^. In addition, alterations in the p53 pathway often represent an alternative route to attenuate the function of WT p53 in tumour^31^,^32^. Here we demonstrate that LED lncRNA is largely silenced in p53 WT primary human ALL. Although our DNA methylation analysis mainly focused on ALL tumours, it is likely that LED inactivation also occurs in other p53 WT tumours, such as on breast, liver and prostate. Nevertheless, this important observation pinpoints the dysregulation of lncRNAs as a potent mechanism in tumorigenesis. In support of this concept, other lncRNAs have been linked with cancer. For example, the oncogenic lncRNA *HOTAIR* is highly expressed in breast tumours and promotes cancer metastasis by guiding polycomb repressive complex 2 to specific genomic loci^28^. The lncRNA ANRIL and SChLAP1 are overexpressed in prostate cancers and antagonize the tumour-suppressive activity of INK4a/b and SWI/SNF complex, respectively^33^,^34^. Finally, tumour-suppressive lncRNAs such as GAS5 have been shown to be downregulated in cancer^35^.

**Figure 6:**
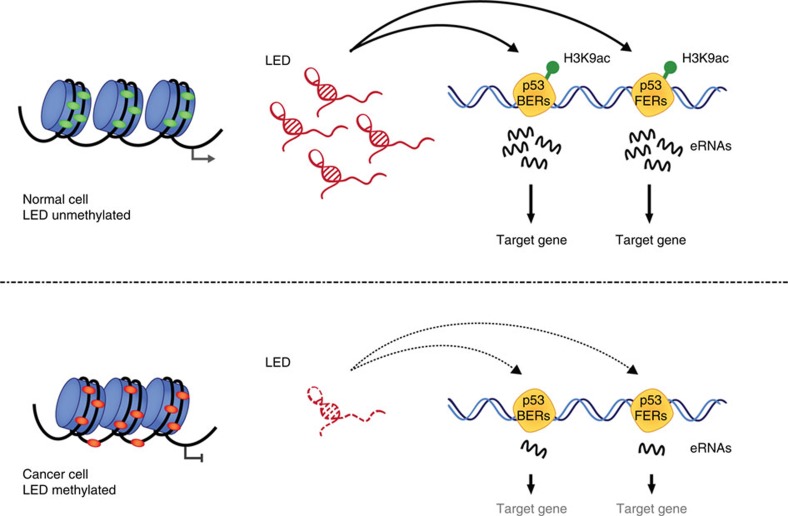
Schematic representation of LED function in normal and cancer cells. P53-bound enhancer regions (p53BERs), p53-free enhancer regions (p53FERs) and H3K9 acetylation are displayed.

Collectively, our results highlight a novel tumour suppressive mechanism involving a p53-induced lncRNA acting on enhancers (Fig. 6). The existence of a crosstalk between different lncRNA species uncovers an emerging regulatory network with potential considerable impacts in cancer development.

## Methods

### Analysis of GRO-seq data and determination of enhancer regions

GRO-seq protocol was performed as previously described^36^. Briefly, MCF-7 cells were incubated with or without 8 μM nutlin-3a for 12 h and 5 million nuclei were isolated for each condition. rRNA reads were removed from the data by alignment to a rRNA index compiled from Ensembl annotations (‘rRNA’, ‘rRNA_pseudogene’ and ‘Mt_rRNA’) using bowtie2 (v.2.0.6, parameters ‘—seed 42 —end-to-end -N1 -L20 -i C,1 -D5 -R5’) and keeping the unmapped reads. GRO-seq data were aligned to hg19 (including unassembled contigs) using bowtie2 (v. 2.0.6) with parameters ‘—seed 42 —sensitive’. Alignments with mapping quality lower than 10 and non-primary alignments were not considered in further analyses. Broad ChromHMM data for nine cell lines^14^ were used to screen putative enhancer regions. Along each chromosome, positions that were marked as enhancer regions (feature IDs 4, 5, 6 and 7) in at least one cell line were merged into regions. Transcription start sites, as annotated by RefSeq (obtained from the UCSC database server, 9 August 2012) and GENCODE v19/BASIC were extended by 1,000 bases and used to blacklist positions (that is, those positions were excluded as putative enhancer regions). Each merged region was tested for the presence of p300 and Pol2 as determined by ENCODE in MCF-7 cells (accessions GSM822295 and GSM1010800 (refs 37, 38)) and each region without p300 and Pol2 peak was removed. The remaining regions were considered putative enhancer regions with enhancer marks. GRO-seq counts were obtained for each region, after extending each region by 1 kb. Regions having detectable transcription on both strands were considered putative enhancer regions with bidirectional transcription and used for downstream analyses. edgeR^39^ was used to determine statistical significance of differential expression of the enhancer regions (separately for each strand).

### Generation of omnibus annotation

Ensembl annotations (v37.65), RefSeq gene annotations (obtained from UCSC database server 9 August 2012) and the Broad Linc RNA catalogue^20^ were merged in a single GTF (annotation file) using the gffread utility supplied with the Cufflinks package (v. 1.3.0), using parameters ‘-M -K -F -G’. This essentially collapses overlapping exons/transcripts so as to create an omnibus with low degree of redundancy yet high coverage of known and novel transcripts.

### Analysis of RNA-seq data

RNA-seq samples were processed according to Illumina’s protocol. Raw RNA-seq data were aligned using TopHat2 (v. 2.0.3)^40^, using parameters ‘-m1 -p4 -F0.0 —segment-length 21 —segment-mismatches 1’ and an exon annotation GTF file that was generated as described before. Reads with mapping quality less than 10 and non-primary alignments were discarded. Remaining reads were counted using HTSeq-count (http://www-huber.embl.de/users/anders/HTSeq/doc/overview.html), per gene ID. Statistical analysis of the differential expression of genes was performed using edgeR^39^. Genes with False Discovery Rate (FDR) for differential expression lower than 0.01 were considered significant.

### Analysis of p53 ChIP data

p53 ChIP-seq data obtained from MCF-7 cells upon untreated and p53-stimulated conditions were obtained from SRA project SRP007261. Alignment was done using bowtie2 (v. 2.0.6) with parameters ‘—seed 42 —sensitive’ to hg19 (including unassembled contigs). Only primary alignments with quality of at least ten were kept. Peaks were called by MACS (v. 2.0.10.20130501) using default parameters. Peaks with a fold-change (w.r.t. input) lower than 3.0 or a -log10(q-value) lower than 2.0 were discarded.

### Sequence motif enrichment analysis

For enhancer regions the midpoint of bidirectional transcription was established, after pooling the GRO-seq data of the nutlin-3a-treated replicates. Two hundred bases of DNA around the midpoint of bidirectional transcription were extracted. For the analysis of sequence enrichment of BERs and FERs, the backgrounds were enhancers with bidirectional transcription that had an FDR for differential expression of 0.75 or greater (for both strands). In addition, for FERs the background contained only enhancers that had no p53 peak within 1 kb. HOMER^17^ was used to search for overrepresented sequence motifs, using parameters ‘-nogo -nlen 4 -len 18 -S 5 -mis 2’.

### Constructs

p21e domain sense and antisense were PCR amplified using gDNA derived from MCF-7 cell lines and subsequently cloned (*Nhe*1/*Xho*1) into pGL3-promoter luciferase reporter vector. All primers used are listed in Supplementary table 1.

### Cell culture and transfection

MCF-7 cells were cultured in DMEM containing 10% FBS, penicillin and streptomycin at 37 °C and 5% CO_2_. Identification and validation of lncRNA regulated by nutlin-3a were carried out by treating MCF-7 cells with 2–8 μM of nutlin-3a for a period of 4–12 h. To induce a p53 stress-response, cells were also treated with 10 Gy of ionizing radiation for 12 h. RNA interference experiments were performed using Dharmafect transfection reagent-1 and between 20 and 60 nM of siRNA. For epigenetic study, cells were treated with 2 μM 5-aza-2′-deoxycytidine (A3656, Sigma) for 72 h.

### Protein analysis

Whole-cell lysates were prepared as previously described^31^. Protein detection was performed using primary antibodies detecting p53 (DO1, Santa Cruz, 1:1,000), p21 (Sc-397, Santa Cruz, 1:1,000), CDK4 (Sc-260, Santa Cruz, 1:1,000), phospho-histone H3 (ser 10) (9701, Cell Signaling, 1:100). Proteins were visualized using adequate secondary antibody (Dako) and ECL reagents (GE Healthcare).

### Quantitative real-time PCR

RNA isolation and cDNA preparation were carried out as previously described^9^. Real-time qPCR analysis was performed using the LightCycler 480 SYBR Green I Master mix (Roche). The glyceraldehyde 3-phosphate dehydrogenase was used as an internal control.

### Flow cytometry

Control or nutlin-3a-treated cells were arrested in mitosis using 250 ng ml^−1^ of nocodazole for 24–36 h. Cells were then trypsinized, washed and resuspended in PBS containing 0.6% NP-40, 50 mg ml^−1^ RNaseA and 50 mg ml^−1^ propidium iodide for 10 min. Cell cycle profiles were captured using FACSCalibur (BD Biosciences) and analysed with the Flowjo software.

### Immunofluorescence

Cells were first plated at a density of 3 × 10^5^ cells per well and concomitantly reverse transfected with a control siRNA or siRNAs against LED or p21. After 24 h, cells were trypsinized and seeded on microscope coverslips coated with polylysine. Next, cells were fixed with 3% formaldehyde and subsequently permeabilized with PBS-Triton X-100 (0.3%) solution. After blocking 1 h with 2% PBS-Milk, cells were successively incubated with the primary antibody phospho-histone H3 (ser 10) and the Alexa Fluor 488 Dye-conjugated secondary antibody. Images were captured using an AxioCam MRc CCD camera (Carl Zeiss Microimaging).

### RNA fluorescence *in situ* hybridization

MCF-7 cells treated with nutlin-3a (8 μM) and non-treated controls were grown on coverslips in six-well plates overnight. The media were aspirated and cells washed 3 × in cold PBS. Fixation solution (5 ml of 10 × PBS, 5 ml of 37% formaldehyde (100% formalin) and 40 ml of Diethylpyrocarbonate (DEPC) treated H_2_O) was added and cells were incubated for 20 min at 4 °C. Cells were washed 3 × in cold PBS and 70% cold ethanol was used to permeabilize cells at 4 °C for 24 h. Cells were washed with cold PBS and left in hybridization buffer (1 g of dextran sulfate, 7 ml of DEPC water, 1 ml of formamide and 1 ml of 20 × SSC buffer) for 1 h. A measure of 50 ng of stellaris probe were used in hybridization buffer and cells were kept incubating for 48 h at 37 °C. After washing with wash SSC buffer, coverslips were covered with Draq5 during 20 min for nuclear staining, washed with cold PBS and mounted using antifade buffer (850 μl of DEPC H_2_O, 100 μl of 20 × SSC, 40 μl of 10% glucose, 10 μl of Tris). Images were captured in a Zeiss confocal microscope.

### ChIRP

ChIRP was performed as previously described^41^. ChIRP probes (48 × 40-mer) targeting LED and lacZ were designed at http://www.singlemoleculefish.com/designer.html. Probes antisense to LED were divided into two sets (odd and even). The input and odd and even probe samples were sequenced individually. After clipping of adapters from the obtained reads, data were aligned to hg19 using bowtie2 (v. 2.0.6) using parameters ‘—seed 42 -N 1 -p 2’. Reads with mapping quality less than 10 and non-primary alignments were excluded from further analysis. Peak detection was run using MACS (v. 2.0.10.20130501)^42^ using parameters ‘ -g hs -B -p 0.1’. Peaks with a −log10(*q*-value) ≥5 and an enrichment ≥4 with respect to the input were kept, and peaks found in the odd and even samples were intersected. Overlapping peaks in both samples that had a position-wise Pearson correlation of abundance ≥0.2 and at least 25 reads in both samples were merged. From the resulting set of peaks, plasmid contaminants were discarded (Supplementary Table 2).

### ChIP

MCF-7 (5 × 10^6^) cells were first transfected with a control or specific siRNAs. Next, cells were fixed with 1% formaldehyde for 8 min at room temperature and subsequently quenched with 125 mM glycine for 5 min on ice. The cells were pelleted 10 min at 470 *g* and re-suspended in 300 μl of cold lysis buffer (50 mM Tris-HCl, pH 8.0, 10 mM EDTA and 1% SDS) supplemented with protease inhibitor cocktail (Roche). The suspension was sonicated 20 min (30 s on/off at maximum power) and further diluted with 800 μl of dilution buffer (10 mM Tris-HCl, pH 7.5, 140 mM NaCl, 1 mM EDTA, 0,5 mM EGTA and 1% Triton X-100) supplemented with protease inhibitor cocktail. The lysate was centrifuged for 10 min at maximum speed and the soluble fraction (chromatin) was transferred to a new tube. For each ChIP reaction, 100 μl of chromatin preparation was diluted with 300 μl of dilution buffer and incubated on an end-to-end rotator with 2–10 μg of antibody at 4 °C overnight. Then, 30 μl of protein A/G beads, previously blocked 1 h with PBS/BSA (0.1%) solution, was add to each ChIP reaction and incubated 2–3 h at 4 °C. The immune-purified chromatin was washed 2 × 5 min with the dilution buffer and 1 × with TE (50 mM Tris-HCl pH 8.0 and 10 mM EDTA) and finally eluted in 300 μl elution buffer (20 mM Tris-HCl pH 7.5, 5 mM EDTA, 50 mM NaCl and 1% SDS) at 65 °C overnight. Eluted chromatin was purified using QIAquick PCR purification kit (Qiagen) and subjected to real-time PCR analysis. Antibodies and amounts used in this study were as follows: pol2 (8 μg, CTD4H8, Upstate), H3K9ac (3 μl, ab4441, Abcam), H3K27ac (3 μl, ab4729, Abcam), H3K4me1 (6 μl, ab8895, Abcam), H3K4me3 (3 μl, MC315, Upstate), histone H3 (5 μl, 2650, Cell Signaling).

### Chromosome conformation capture on chip (4C)

4C templates preparation and analysis were performed as previously described^43^. Briefly, 10^7^ of MCF-7 cells were harvested and crosslinked with formaldehyde 2% for 10 min at room temperature, and neutralized with 125 M glycine. After washing with PBS, cells were lysed in 150 mM NaCl, 50 mM Tris-HCl (ph 7.5), 5 mM EDTA, 0.5% NP40, 1% Triton X-100 and nuclei were recovered by spinning 8 min at 600 *g*. Nuclei were digested overnight with 400 U *Dpn*II (NEB) and *Csp*6I (NEB) and re-ligated in 7 ml with 100 U of T4 DNA ligase (Roche) overnight at 16 °C. Purified DNA circles were digested with 50 U of *Dpn*II (Csp6I circles) and *Csp*6I (DpnII circles) overnight at 37 °C, followed by heat inactivation and ligation in 14 ml with 200 U of T4 DNA ligase. The 4C template was then purified and used for PCR amplification.

### DNA methylation analysis

The Methyl Primer Express v1.0 software was used to identify the CpG islands and design-specific primers for the methylation analysis (Supplementary table 1). DNA methylation status was established by bisulfite genomic sequencing of multiple clones or methylation-specific PCRs in DNA samples previously treated with sodium bisulfite (EZ DNA methylation Gold kit, Zymo Research). The Illumina 450 K methylation array was used to anlyse the methylation status in multiple human cancer cell lines. For epigenetic drug treatments, cells were treated with 1 μM 5-aza-2′-deoxycytidine (Sigma) for 72 h.

## Author contributions

N.L., C.A.M., K.R. and R.A. designed the research; N.L. and C.A.M. performed the experiments; K.R. performed the data analysis; A.D.-L. and M.E. performed the methylation studies; S.A.M. and R.K. performed the Northern blots; G.K. and R.L. performed the GRO-sequencing experiment; F.A.M., M.L.d.B., A.D.-L. and M.E. contributed to the patient collection samples; A.R.M. helped with flow cytometry experiments; P.J.W., G.G. and W.deL. performed and analysed the 4C experiments; R.A. supervised the project; N.L., C.A.M., K.R. and R.A. wrote the manuscript.

## Additional information

**Accession codes:** The sequencing data have been deposited with the GEO under accession number GSE53499.

**How to cite this article:** Léveilleé, N. *et al*. Genome-wide profiling of p53-regulated enhancer RNAs uncovers a subset of enhancers controlled by a lncRNA. *Nat. Commun.* 6:6520 doi: 10.1038/ncomms7520 (2015).

## Supplementary Material

Supplementary Information Supplementary Figures 1-5, Supplementary Tables 1-2.

Supplementary Dataset 1 Nutlin-3a-responsive lncRNAs identified by RNA-seq analysis.

Supplementary Dataset 2 Putative LED-associated loci determined by ChIRP-seq analysis.

## Figures and Tables

**Table 1:** Association of LED CpG island hypermethylation with TP53 mutational status in human cancer cell lines.

**Methylation**	**TP53 WT**	**TP53 mut**
Methylated	29 (60%)	30 (34%)
Unmethylated	19 (40%)	57 (66%)
Total (135)	48	87

LED, LncRNA activator of Enhancer Domains; mut, mutant; WT, wild type.

**Table 2:** Frequency of LED promoter associated-hypermethylation (M) in cancer patients.

**Tumour type**	**Methylation**	**Total**	***%***
Acute lymphocytic leukaemia	21	95	22
Cutaneous lymphomas	1	6	16
Folicular lymphomas	1	10	10
Melanomas	4	46	9
Acute myeloid leukaemia	1	13	8
Chronic lymphocytic leukaemia	1	33	3

LED, LncRNA activator of Enhancer Domains.
