# Supplementary material for: Genome-wide profiling of p53-regulated enhancer RNAs uncovers a subset of enhancers controlled by a lncRNA
Source: Nat Commun. 2015 Mar 27;6:6520. doi: 10.1038/ncomms7520 (PMC4389233; doi:10.1038/ncomms7520)
Supplement: Supplementary Information — Supplementary Figures 1-5, Supplementary Tables 1-2. [file ncomms7520-s1.pdf]

a

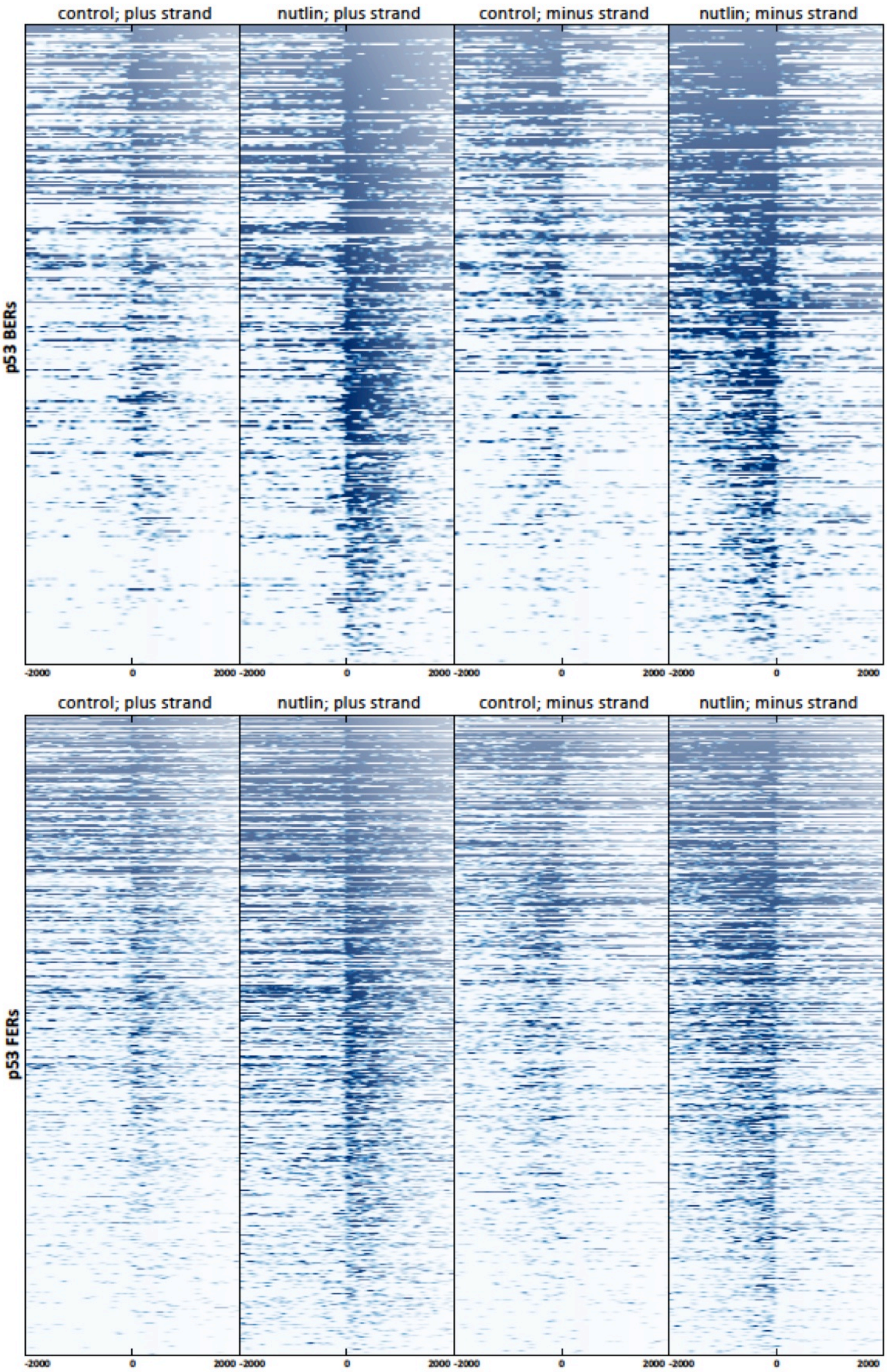
control; plus strand
nutlin; plus strand
control; minus strand
nutlin; minus strand
p53 BERs
p53 FERs
-2000
0
2000

b

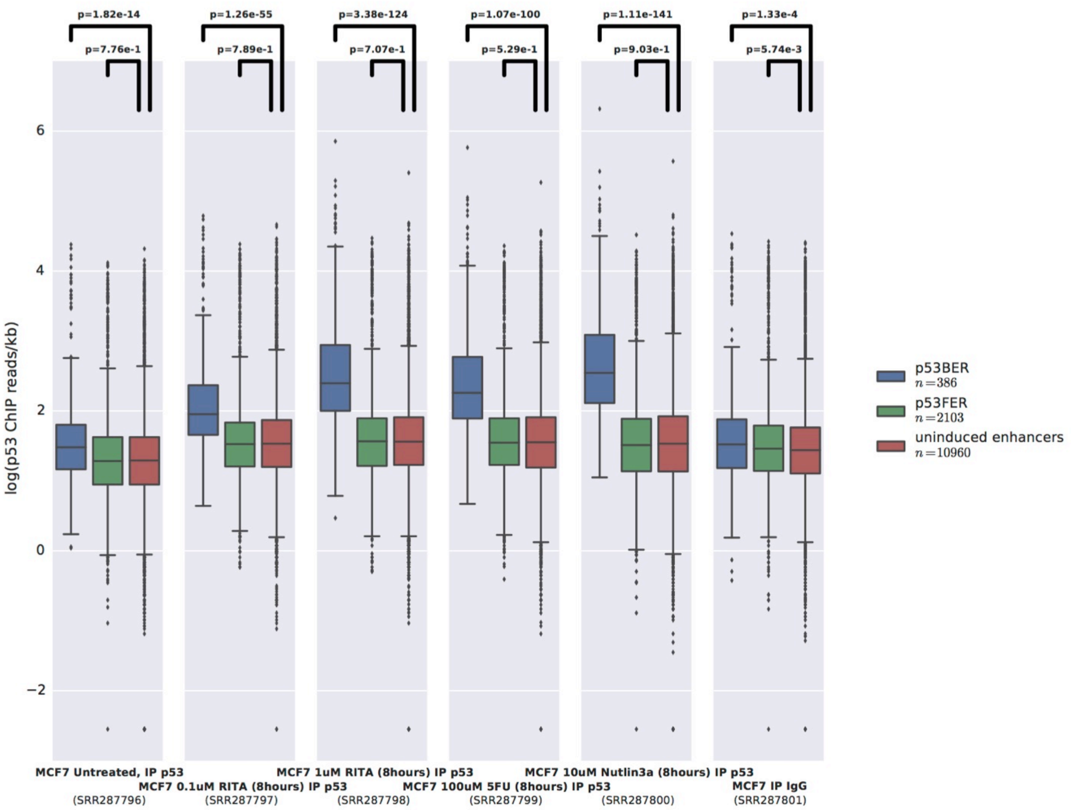
p=1.82e-14
p=7.76e-1
p=1.26e-55
p=7.89e-1
p=3.38e-124
p=7.07e-1
p=1.07e-100
p=5.29e-1
p=1.11e-141
p=9.03e-1
p=1.33e-4
p=5.74e-3
log(p53 ChIP reads/kb)
6
4
2
0
−2
MCF7 Untreated, IP p53 (SRR287796)
MCF7 0.1uM RITA (8hours) IP p53 (SRR287797)
MCF7 1uM RITA (8hours) IP p53 (SRR287798)
MCF7 100uM 5FU (8hours) IP p53 (SRR287799)
MCF7 10uM Nutlin3a (8hours) IP p53 (SRR287800)
MCF7 IP IgG (SRR287801)
p53BER $n=386$
p53FER $n=2103$
uninduced enhancers $n=10960$

c

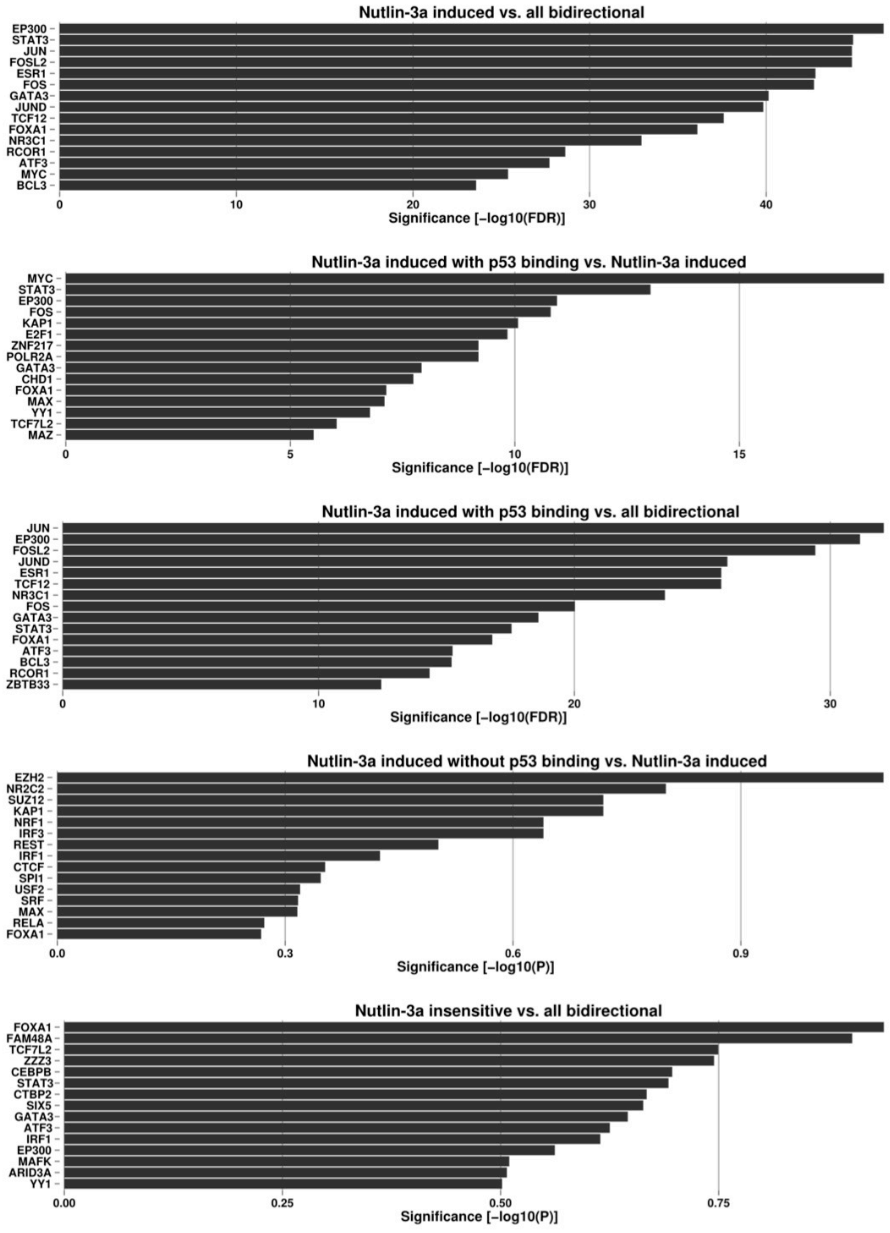
Nutlin-3a induced vs. all bidirectional
EP300
STAT3
JUN
FOSL2
ESR1
FOS
GATA3
JUND
TCF12
FOXA1
NR3C1
RCOR1
ATF3
MYC
BCL3
0
10
20
30
40
Significance [−log10(FDR)]
Nutlin-3a induced with p53 binding vs. Nutlin-3a induced
MYC
STAT3
EP300
FOS
KAP1
E2F1
ZNF217
POLR2A
GATA3
CHD1
FOXA1
MAX
YY1
TCF7L2
MAZ
0
5
10
15
Significance [−log10(FDR)]
Nutlin-3a induced with p53 binding vs. all bidirectional
JUN
EP300
FOSL2
JUND
ESR1
TCF12
NR3C1
FOS
GATA3
STAT3
FOXA1
ATF3
BCL3
RCOR1
ZBTB33
0
10
20
30
Significance [−log10(FDR)]
Nutlin-3a induced without p53 binding vs. Nutlin-3a induced
EZH2
NR2C2
SUZ12
KAP1
NRF1
IRF3
REST
IRF1
CTCF
SPI1
USF2
SRF
MAX
RELA
FOXA1
0.0
0.3
0.6
0.9
Significance [−log10(P)]
Nutlin-3a insensitive vs. all bidirectional
FOXA1
FAM48A
TCF7L2
ZZZ3
CEBPB
STAT3
CTBP2
SIX5
GATA3
ATF3
IRF1
EP300
MAFK
ARID3A
YY1
0.00
0.25
0.50
0.75
Significance [−log10(P)]

d

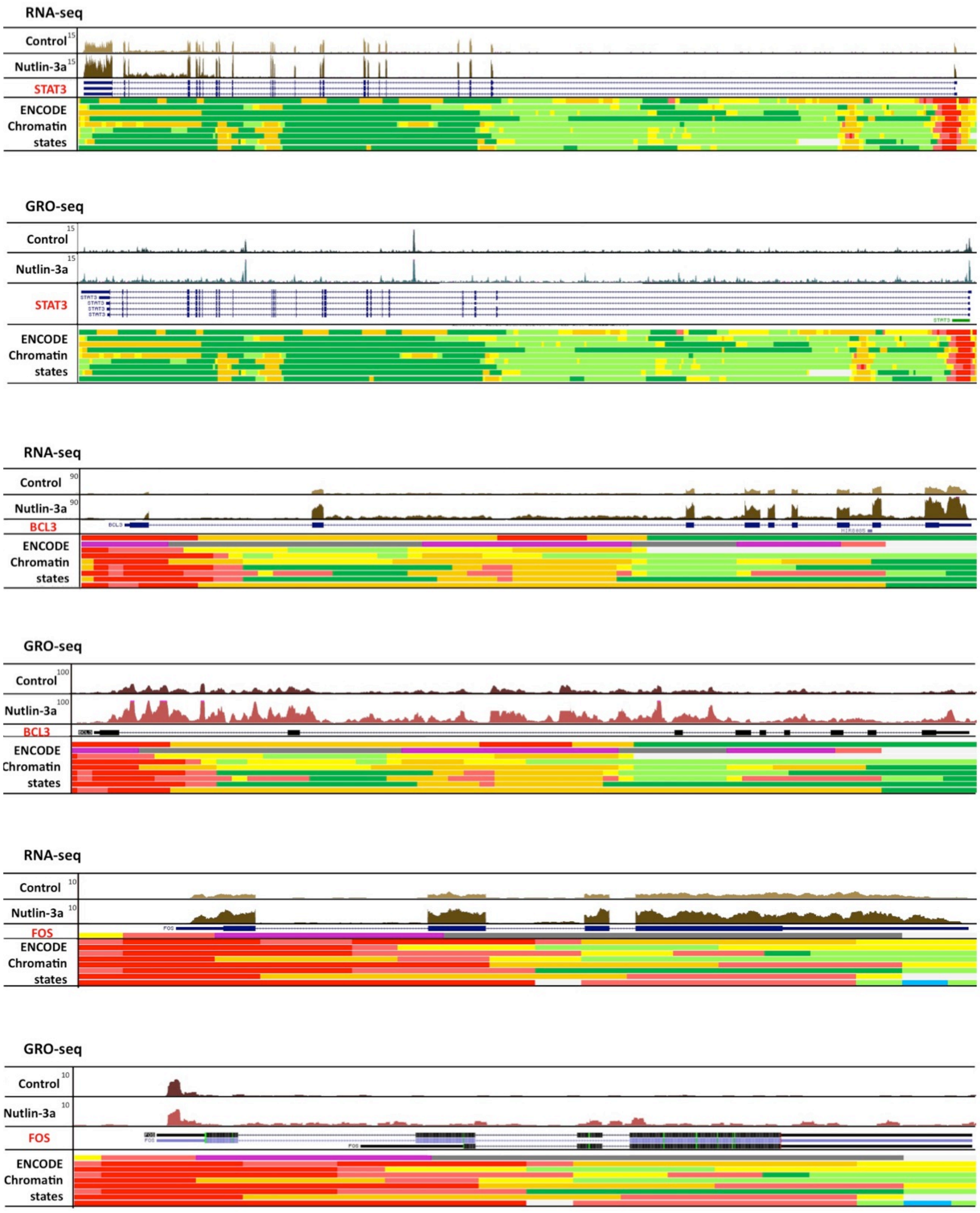
RNA-seq
Control
Nutlin-3a
STAT3
ENCODE Chromatin states
GRO-seq
Control
Nutlin-3a
STAT3
ENCODE Chromatin states
RNA-seq
Control
Nutlin-3a
BCL3
ENCODE Chromatin states
GRO-seq
Control
Nutlin-3a
BCL3
ENCODE Chromatin states
RNA-seq
Control
Nutlin-3a
FOS
ENCODE Chromatin states
GRO-seq
Control
Nutlin-3a
FOS
ENCODE Chromatin states

e

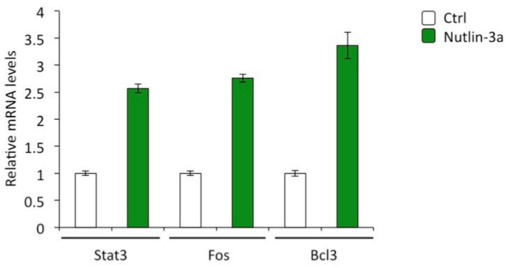

f

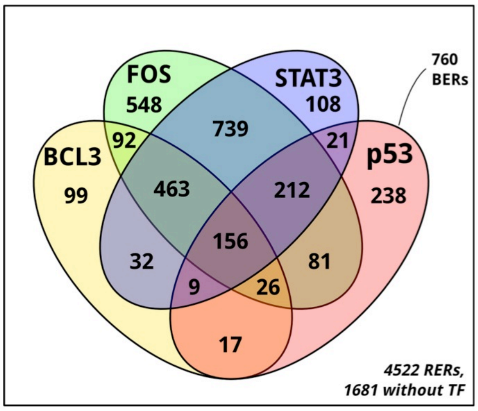

Supplementary Figure 1. Identification of p53-regulated enhancer RNAs (p53RERs). (a) GRO-seq read densities centered at the point of bidirectional transcription for p53BERs (upper panel) and p53FERs (lower panel). (b) Boxplot showing publically available p53 ChIP-seq coverage for p53BERs, p53FERs and uninduced enhancers regions. P-values are for the 2-sided Mann-Whitney-U test. (c) Top 15 transcription factor enrichments. Top to bottom: p53RERs vs all bidirectional enhancers, p53BERs vs. p53RERs, p53BERs vs. all bidirectional, p53FERs vs. p53RERs, nutlin-3a-insensitive vs. all bidirectional (as negative control). Note that in the last two panels the significance is indicated by uncorrected p-value, while in the other panels the FDR is used. (d) Genome browser representation of STAT3, FOS and BCL3 activation upon nutlin-3a treatment in MCF-7 cells, shown by both RNA-seq and GRO-seq. (e) Relative mRNA levels of STAT3, FOS and BCL3 upon nutlin-3a (8 µM) treatment in MCF-7 cells. Values were determined by qRT-PCR (Mean ± SD are shown). (f) Venn diagram showing the number of p53-responsive enhancers (from the total of 4522, see Fig. 1c in the main text) that have binding of transcription factors BCL3, FOS, STAT3 and p53. The binding positions of BCL3, FOS and STAT3, as determined by ChIP-seq, were obtained from ENCODE data (wgEncodeReg TFBS Clustered V3).

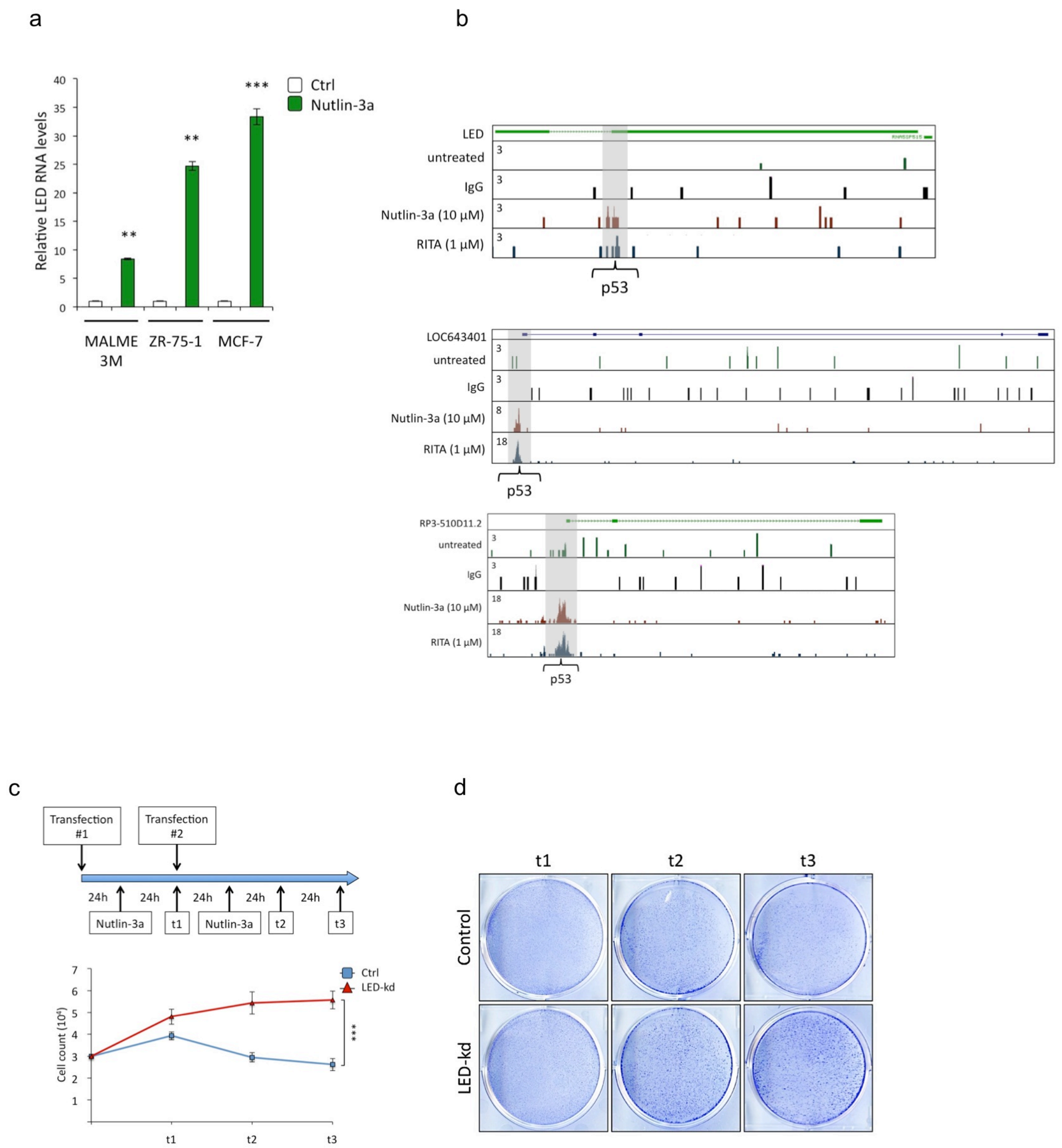

Supplementary Figure 2. Novel stress-regulated lncRNA LED. (a) LED expression levels in MALME-3M, ZR-75-1 and MCF-7 cells upon 12h nutlin-3a treatment (8 µM). Values were determined by qRT-PCR (n=3; p-values were calculated with a two-tailed student's t-test. **p<0.01, ***p<0.005). (b) P53 or IgG ChIP in MCF-7 cells treated with or without nutlin-3a (8 µM) and RITA (1µM) treatments. The binding of p53 within LED gene body is displayed. (c) Schematic representation of cell proliferation assay in MCF-7 treated with 2 pulses of nutlin-3a and transfected with a control or LED siRNA. (n=3; p-values were calculated with a two-tailed student's t-test. ***p<0.005). (d) Colony formation assay using the same condition as in (c).

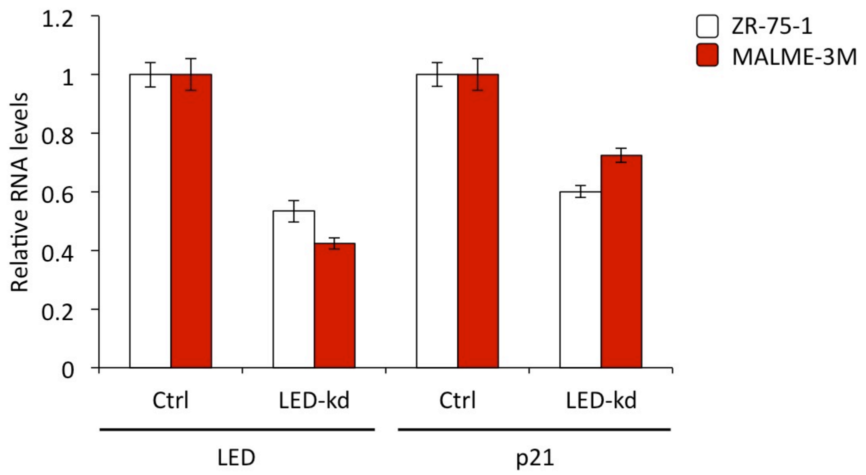

Supplementary Figure 3. The lncRNA LED influences p21 levels. LED and p21 expression levels in ZR-75-1 and MALME-3M cell lines upon transfection of a control (Ctrl) or LED siRNA (LED-kd). The cells were treated 12h with nutlin-3a (8 μM) and expression levels were measured by qRT-PCR (Mean ± SD are shown).

SUPPLEMENTARY FIGURE 4

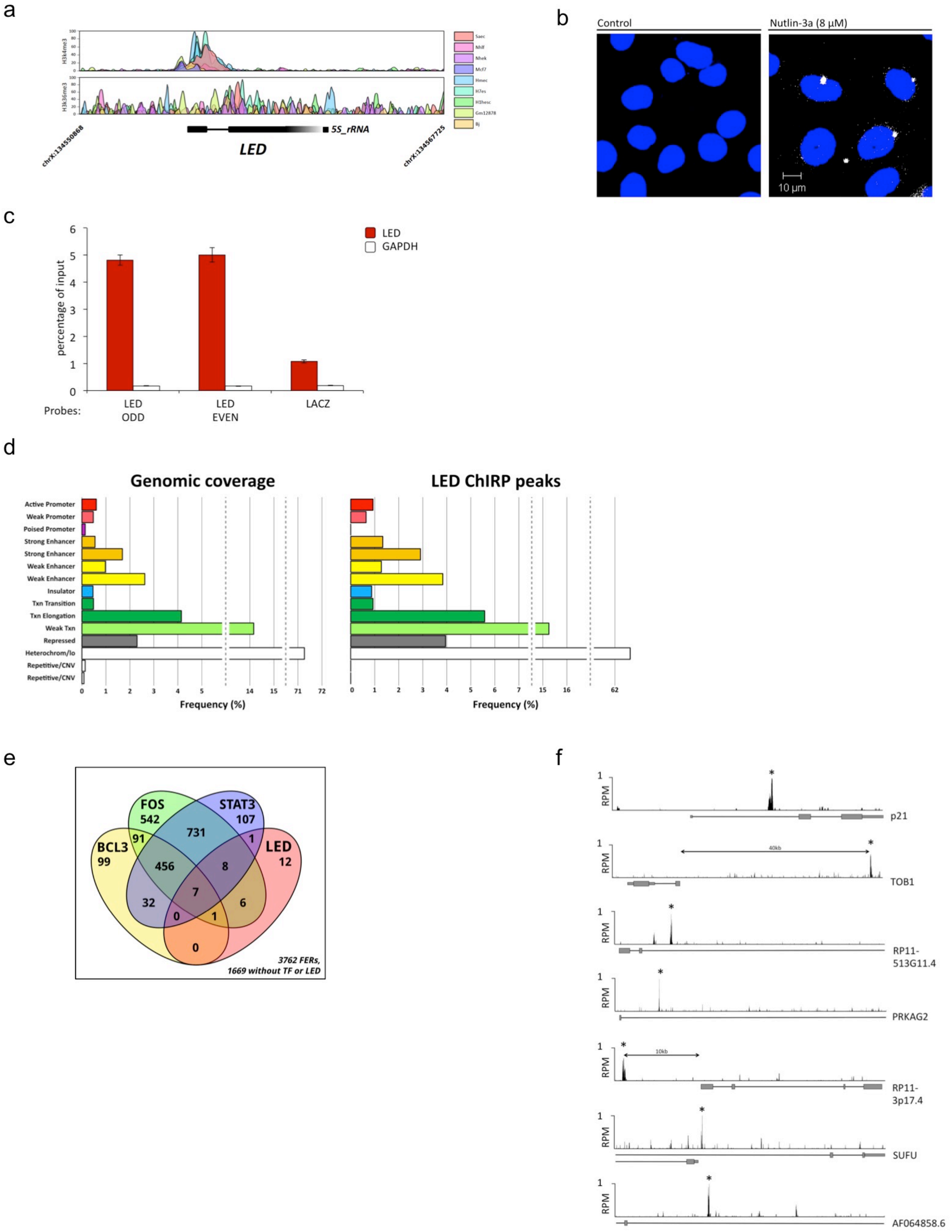

g

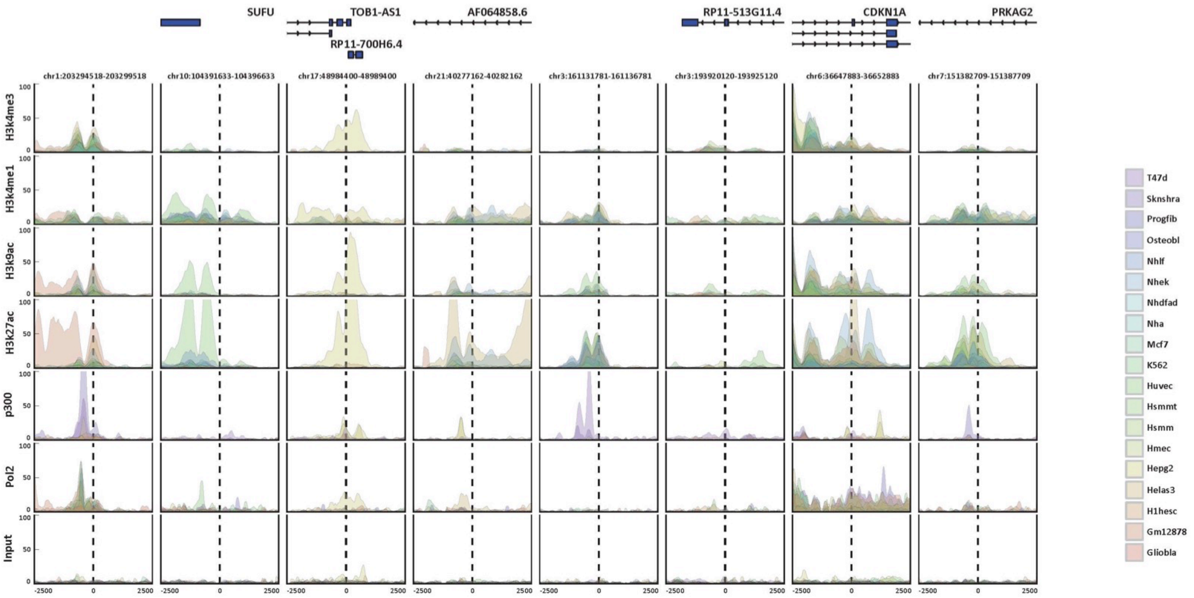
SUFU
TOB1-AS1
RP11-700H6.4
AF064858.6
RP11-513G11.4
CDKN1A
PRKAG2
chr1:203294518-203299518
chr10:104391633-104396633
chr17:48984400-48989400
chr21:40277162-40282162
chr3:161131781-161136781
chr3:193920120-193925120
chr6:36647883-36652883
chr7:151382709-151387709
H3k4me3
H3k4me1
H3k9ac
H3k27ac
p300
Pol2
Input
-2500
0
2500
T47d
Sknshra
Progfib
Osteobl
Nhlf
Nhek
Nhdfad
Nha
Mcf7
K562
Huvec
Hsmmt
Hsmm
Hmec
Hepg2
Helas3
H1hesc
Gm12878
Gliobla

h

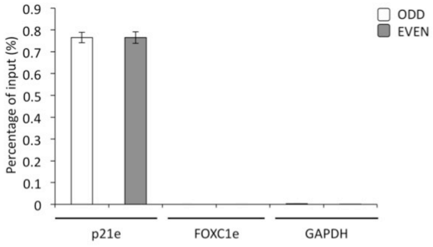
ODD
EVEN
Percentage of input (%)
0.9
0.8
0.7
0.6
0.5
0.4
0.3
0.2
0.1
0
p21e
FOXC1e
GAPDH

i

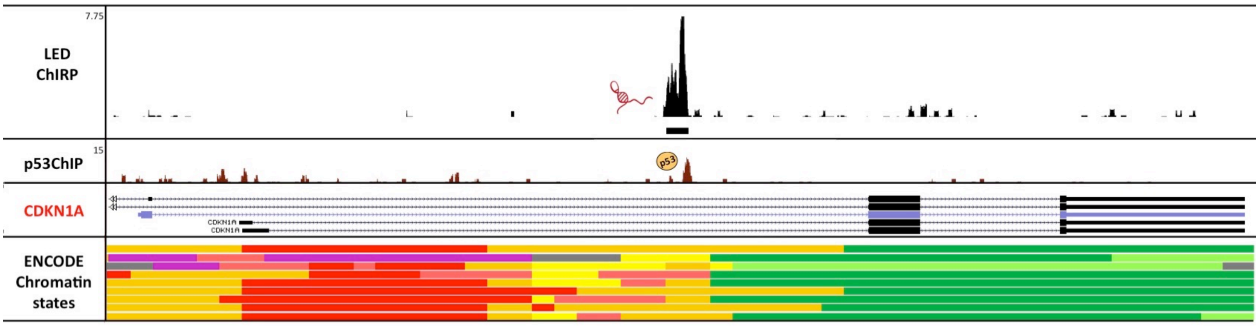
LED ChIRP
7.75
p53ChIP
15
p53
CDKN1A
ENCODE Chromatin states

j

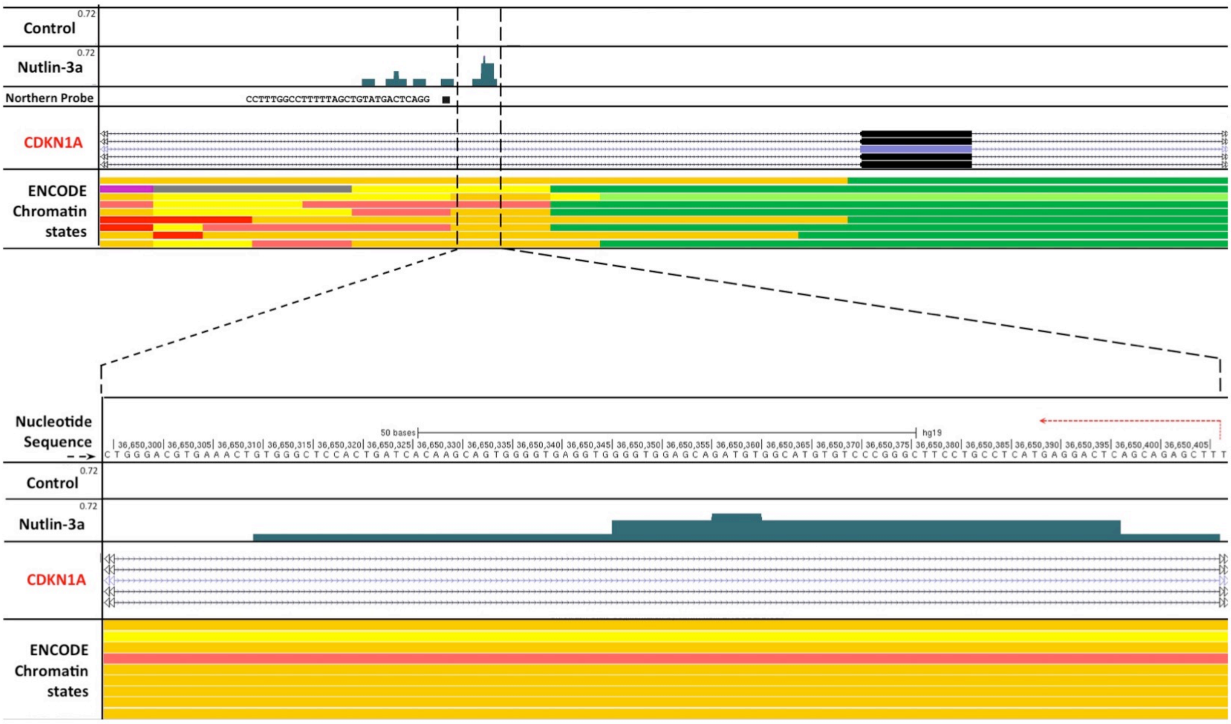
Control
Nutlin-3a
Northern Probe
CCTTTGGCCTTTTTAGCTGTATGACTCAGG
CDKN1A
ENCODE
Chromatin
states
Nucleotide
Sequence
50 bases
hg19
Control
Nutlin-3a
CDKN1A
ENCODE
Chromatin
states

k

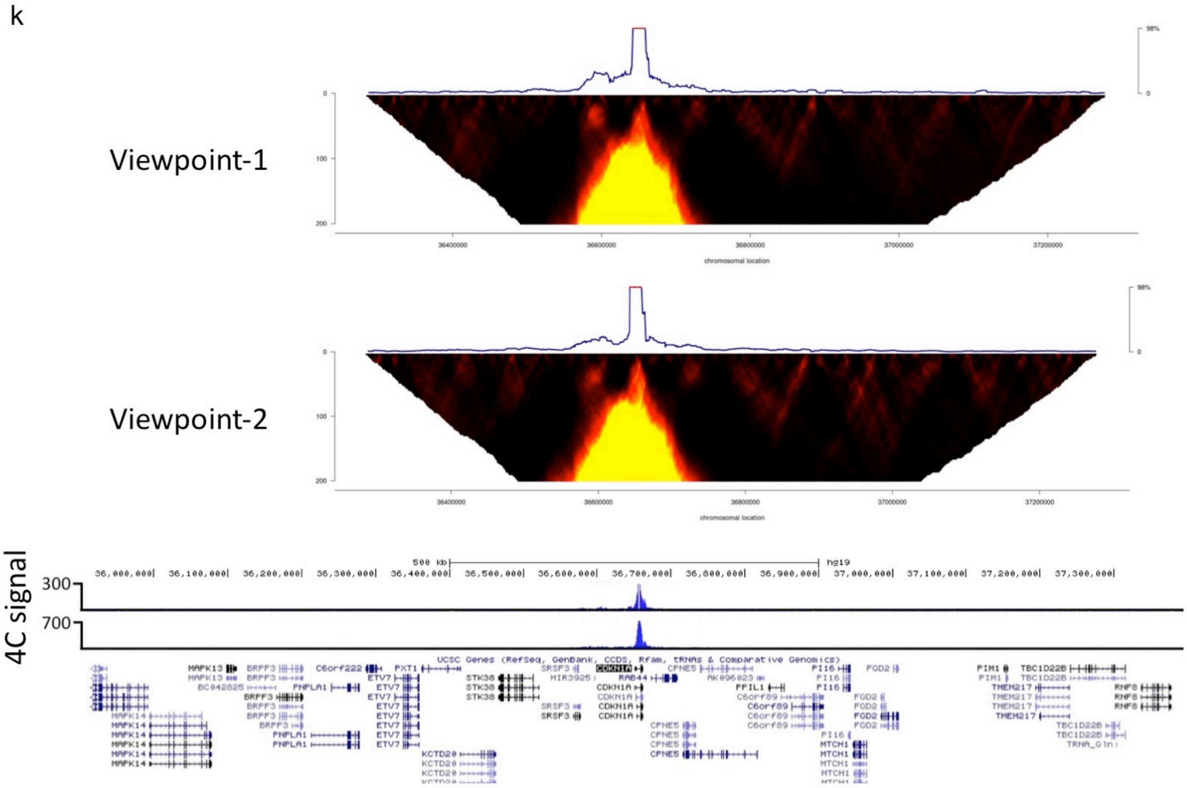
Viewpoint-1
Viewpoint-2
chromosomal location
500 kb
hg19
4C signal
300
700
UCSC Genes (RefSeq, GenBank, CCDS, Rfam, tRNAs & Comparative Genomics)

l

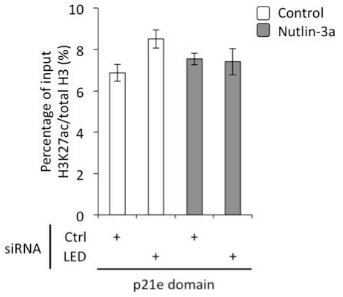

m

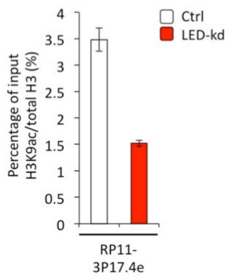

n

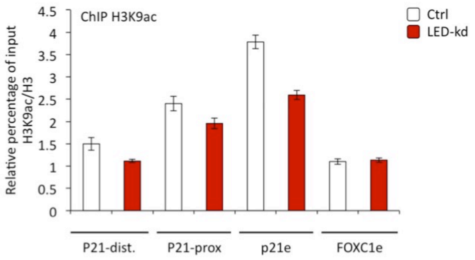

o

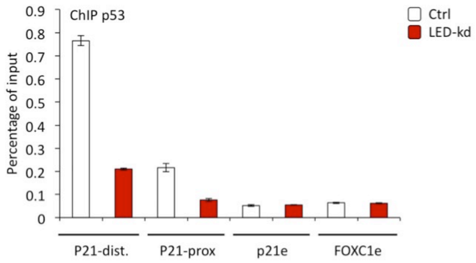

Supplementary Figure 4. LED binds preferentially to enhancers and regulates enhancer RNA production from p53RERs. (a) H3K4 trimethylation and H3K36 trimethylation (K4K36 signature) at LED gene locus for several cell lines, as determined by the ENCODE consortium. (b) RNA Fluorescence *in situ* hybridization (FISH), with probes against LED in MCF-7 cells incubated with or without nutlin-3a. (c) ChIRP enrichment for LED RNA using LED-ODD, LED-EVEN and LACZ probes. GAPDH was used as a control (mean ± SD). (d) Distribution of the ENCODE features over the genome (left panel) and LED ChIRP peaks in the different ENCODE features (right panel). (e) Intersection of LED-bound p53FERs with BCL3, FOS and STAT3 transcription factors. (f) ChIRP sequencing data, showing LED association to a subset of enhancer domains. Values are represented by RPM (reads per million). Star (*) indicates the significant binding site. (g) Levels of several histone marks (H3K27Ac, H3K4me1, H3K4me3 and H3K9Ac), p300 and RNAPII in different cell lines in the loci (+/- 5kb) of selected LED-induced enhancers, as determined by the ENCODE consortium. (h) DNA ChIRP-qPCR enrichment for p21e, but not for FOXC1e or GAPDH, using ODD and EVEN LED-tiling probes in MCF-7 cells. Values are represented as percentage of input. Mean ± SD are shown. (i) Genome browser depicting the overlapping LED-binding site (ChIRP) with a p53BS (ChIP) at p21 intronic enhancer (p21e). (j) Genome browser session showing p21 enhancer domain (p21e) and the putative p21 enhancer RNA transcription start site (red arrow). Control and nutlin-3a-treated MCF-7 cells GRO-seq tracks are shown as well as the "+" strand nucleotide sequence. (k) Domainograms (de Wit et al., 2008) visualizing significance of interactions at different window sizes (viewpoints) for p21 enhancer domain with surrounding chromosomal regions in MCF-7 cells. Color ranges (see scale bar) reflect different levels of significance, from black (low significance, P=1x10-2) to yellow (high significance, P=1x10-8). To account for the fact that the majority of the data are

very close to the viewpoint, we set the data range of the vertical axis to the 98% quantile value for the analyzed region. Values are represented by intensity of 4C signal. Schematic representation of p21 gene with the location of the bait used for the 4C experiments is also shown. (l) ChIP-qPCR for total H3 and H3K27Ac at p21 enhancer domain (p21e) in control and nutlin-3a-treated MCF-7 cells transfected with a control or LED siRNAs (LED-kd). Values represent the percentage of input and were normalized to total H3. Mean ± SD are shown. (m) ChIP-qPCR for total H3 and H3K9Ac at RP11-3P17.4e in nutlin-3a-treated MCF-7 cells transfected with a control or LED siRNA (LED-kd). Values represent the percentage of input and were normalized to total H3. Mean ± SD are shown. (n) ChIP-qPCR for total H3 and H3K9Ac at p21 distal (dist.), proximal (prox.) and intronic (p21e) enhancers in nutlin-3a-treated MCF-7 cells transfected with a control or LED siRNA (LED-kd). Values represent the percentage of input and were normalized to total H3. Mean ± SD are shown. (o) ChIP-qPCR for p53 at p21 distal (dist.), proximal (prox.) and intronic (p21e) enhancers in nutlin-3a-treated MCF-7 cells transfected with a control or LED siRNA (LED-kd). Values represent the percentage of input. Mean ± SD are shown.

SUPPLEMENTARY FIGURE 5

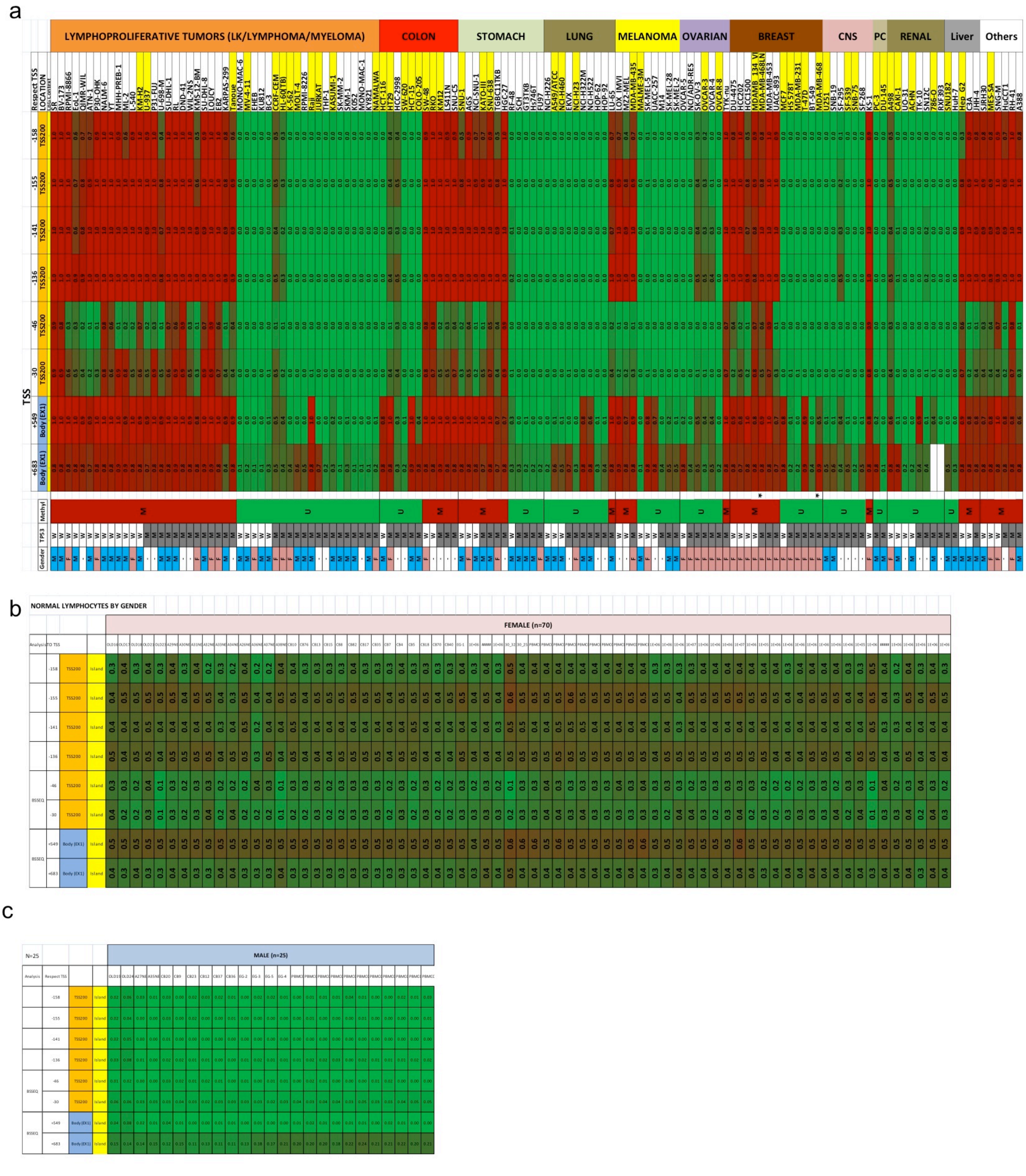

d

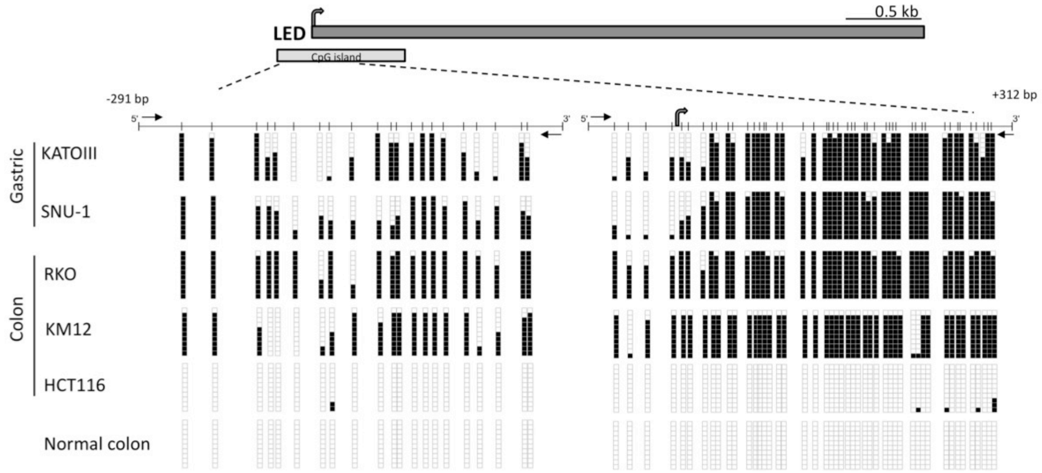
LED
0.5 kb
CpG island
-291 bp
+312 bp
Gastric
KATOIII
SNU-1
Colon
RKO
KM12
HCT116
Normal colon

e

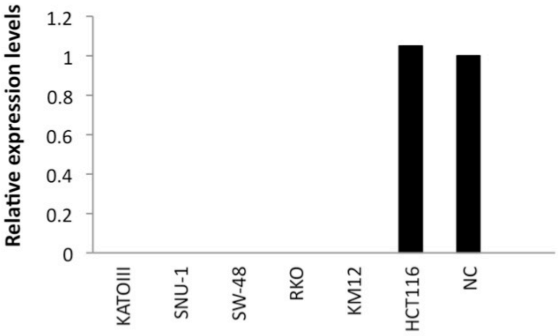
Relative expression levels
1.2
1
0.8
0.6
0.4
0.2
0
KATOIII
SNU-1
SW-48
RKO
KM12
HCT116
NC

f

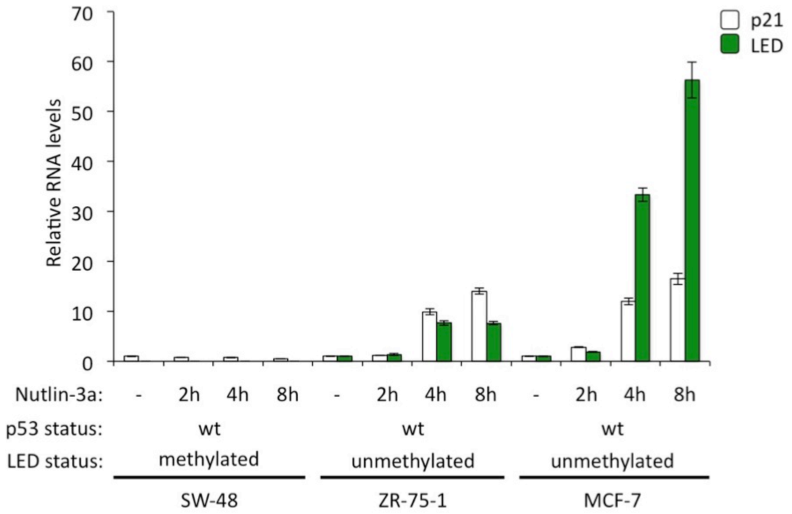
p21
LED
Relative RNA levels
70
60
50
40
30
20
10
0
Nutlin-3a: - 2h 4h 8h - 2h 4h 8h - 2h 4h 8h
p53 status: wt wt wt
LED status: methylated unmethylated unmethylated
SW-48
ZR-75-1
MCF-7

Supplementary Figure 5. DNA methylation-associated silencing of LED in colon and gastric cancers. (a) DNA methylation-associated silencing of LED in several cancer cell lines. Methylation was measured in different LED regions represented by TSS (transcription start site: -158, -155, -141, -136, -46, -30, +549, +683). Presence of unmethylated or methylated CpGs is indicated by green or red, respectively. Methylation is considered when both CpGs at (-155, -141, -136) and at (+549,+683) are methylated or when all the CpGs are methylated. Indication of TP53 status (W-wild type ; M-mutant) and gender (M-male ; F-female) are represented. (b-c) LED methylation in normal lymphocytes by gender- female (b) and male (c) and analysed as in (a). (d) Schematic representation of LED gemomic loci and CpG island. Bisulfite genomic sequencing analysis of LED CpG island in human solid cancer cell lines and normal colon as as tissue control. Location of bisulfite genomic sequencing PCR primers (black arrows), CpG dinucleotides (vertical lines) and the transcriptional start site (grey arrow) are shown. Ten single clones are represented for each sample. Presence of unmethylated or methylated CpGs is indicated by white or black squares, respectively. (e) LED expression levels in methylated or unmethylated human solid cancer cell lines and in normal colon (NC) as control. Values were determined by qRT-PCR in triplicates. (f) LED and p21 expression levels in SW-48, ZR-75-1 and MCF-7 cells upon 2h, 4h and 8h of nutlin-3a treatment (8 μM). Values were determined by qRT-PCR and normalized to the untreated samples. Mean ± SD are shown.

SUPPLEMENTARY TABLE 1

**Primers**	**5'--> 3'**
**Metylation**	
LED-Bisulfite seq-Fw 1	ATTTTGGGAAATGGTTTTAAAG
LED-Bisulfite seq-Rev 1	CCCAAACCAACATAAATCC
LED-Bisulfite seq-Fw 2	ATTTATGTTGGTTTGGG
LED-Bisulfite seq-Rev 2	ATTCCCTCCTTCTAATTTC
LED-MSP-M-Fw	GGTACGTCGAGTAGGCGGGGTC
LED-MSP-M-Rev	CTACGAAAAACCACCCCCCCG
LED-MSP-U-Fw	GGGGTATGTTGAGTAGGTGGGGTT
LED-MSP-U-Rev	ACTACAAAAAACCACCCCCCCAA
**QPCR**	
QPCR-LED-Fw	TTCTGCTGCGGCGACTTGAC
QPCR-LED-Rev	CTTCTTCTCCTCTTGTTCAG
QPCR-p21-Fw	TACCCTTGTGCCTCGCTCAG
QPCR-p21-Rev	GAGAAGATCAGCCGGCGTTT
QPCR-p21e-Fw	ATGAGACTTGCCCCTGAGTCATAC
QPCR-p21e-Rev	TAACCTCTATCCACCCTCTGCTAG
QPCR-TOB1e-Fw	GAAGTTTGTTTTGGACAGTGTTTG
QPCR-TOB1e-Rev	AGTCTCTCCATAAAGCCTGATGTT
QPCR-RP11-3P17.4e-Fw	TTCTTGTGGTAGGAAGGAGGTAAC
QPCR-RP11-3P17.4e-Rev	CCAGAAGGAAATAGAGCAGAAAAC
QPCR-RP11-513G11e-Fw	TTTTCAGTACGACTAGCTCCTGTG
QPCR-RP11-513G11e-Rev	CGAGGAAAATAATAGCCACATCTT
QPCR-AF064858.6e-Fw	AAAAATCACAGTTCAGCAACAGTC
QPCR-AF064858.6e-Rev	TGCTTAAGTGTCATCATTTTCGTT
QPCR-PRKAG2e-Fw	AAGACTTGGCACTTTGGGTTT
QPCR-PRKAG2e-Rev	GTCTCCTATTGAATTGGTGTCCTT
QPCR-SUFUe-Fw	TAGGAGATATTACAGATGGCATGG
QPCR-SUFUe-Rev	AGAGAATGCAGAATAGCAAGATCC
QPCR-FOXC1e-Fw	CATGAAAGGTGAAGCGGAAATAC
QPCR-FOXC1e-Rev	TGAAGGAGCAGGTGAAACG
QPCR-GAPDH-Fw	TGCACCACCAACTGCTTAGC
QPCR-GAPDH-Rev	GGCATGGACTGTGGTCATGAG
QPCR-LOC643401-Fw	CCAACAATCTCTGGCTGCA
QPCR-LOC643401-Rev	CACATTACCACAGGAGCTGA
QPCR-RP3-510D11.2-Fw	CCAGACCGACGGGACAGCG
QPCR-RP3-510D11.2-Rev	GCTTCCCTGTCCTCCTCCTA
QPCR-S14-Fw	GGCAGACCGAGATGAATCCTC
QPCR-S14-rev	CAGGTCCAGGGGTCTTGGTCC
QPCR-U2-Fw	CATCGCTTCTCGGCCTTTTG
QPCR-U2-rev	TGGAGGTACTGCAATACCAGG
QPCR-STAT3-Fw	CCCATACCTGAAGACCAAGTTTAT
QPCR-STAT3-Rev	TATTTCCAAACTGCATCAATGAAT
QPCR-FOS-Fw	CTGGTGCATTACAGAGAGGAGAA
QPCR-FOS-Rev	GTGTTTCACGCACAGATAAGGTC

QPCR-BCL3-Fw	AACACCGAGTGCCAAGAAAC
QPCR-BCL3-Rev	ACCATGCTAAGGCTGTTGTTTT
**Chip**	
p21-proximal-Fw	GAGGAAGAAGACTGGGCATGTCT
p21-proximal-Rev	GCTTGGAGCAGCTACAATTACTGAC
p21-distal-Fw	GTGGCTCTGATTGGCTTTCTG
p21-distal-Rev	CTGAAAACAGGCAGCCCAAG
MDM2 prom-Fw	GGGCTATTTAAACCATGCATTTTC
MDM2 prom-Rev	GTCCGTGCCCACAGGTCTA
LED-p53site-Fw	CCCATCTCTTTCTTTCTCTTTCAG
LED-p53site-Rev	CTGGGCAGGGATGTGACTCT
**siRNAs**	
LED-kd1	CCUCGGUGAUGCUUAACGUC
LED-kd2	CUUCCCCUACUUCUACAUCG
RP3-510D11.2-KD2	CUCCACCUGCGCAAAACGAA
RP3-510D11.2-KD3	GGGCGGAUUCUCCUUGGACU
RP3-510D11.2-KD4	GCGGCCAUCAGUAACAGCAA
LOC643401-KD1	CAGCUCCUGUGGUAAUGUGG
LOC643401-KD2	UCACCAGGGUGAGUGAACAA
LOC643401-KD3	GUGUGAGGUGGUGCUAUGUA
LOC643401-KD4	AGGAGCACAAUUCUGCAACA
p53-kd	GACUCCAGUGGUAAUCUAC
**Cloning**	
P21 enhancer-Nhe1-Fw	GACGACGCTAGCTAGTTGCCCAGGCTAGTCTTGGAC
P21 enhancer-Xho1-rev	GACGACCTCGAGTATAGAGAAGTGCATTGAAAACAG
P21 enhancer-Xho1-R-Fw	GACGACCTCGAGTAGTTGCCCAGGCTAGTCTTGGAC
P21 enhancer-Nhe1-R-rev	GACGACGCTAGCTATAGAGAAGTGCATTGAAAACAG
pGL3basic-LEDp53BS-Fw	CGGCTAGCCGCCGGGGGAAGCAAGGACGTG
pGL3basic-LEDp53BS-Rev	CCTCGAGGCCAGGGAACTCAGACCTTCC
**Chirp**	**modified with C3(biotin)**
LED-1	GTAGTTTCTGGAGCCCGATG
LED-2	CCCTCTTTGGCCAATATGAG
LED-3	CAAATGCCAATGCCTTTCTG
LED-4	GGGAACTCAGACCTTCCTTC
LED-5	CAAGGAAAGCGACATTCCTA
LED-6	GATCTTCCCTGTGACTTTCC
LED-7	AGCATTGTACACCAGTTAGC
LED-8	GTTTTCAGTCCCAGGAACTG
LED-9	CAGCTTCATTCAGGACCTGA
LED-10	TGTCACTCTTGTCCCATAGA
LED-11	ATCTGTGCATCAGACACAAG
LED-12	TGACACCAGGGTTTGAAAGG
LED-13	CTCAGCAAAGAGCTTTCCTC
LED-14	CGCAACTTTATCCCAGTAGG

LED-15	GACTTCCGTGACCCTCTTAT
LED-16	ACCGCCTAGAAAACAGACTG
LED-17	GTATGTCATTCCGGTCAGTG
LED-18	ACACAGTGAATCACACACAG
LED-19	AAACAAGAACAGCTCTCTGG
LED-20	ACAAGAAGACGTGACAACAC
LED-21	GCCTCCAGTTGGGATTTTAG
LED-22	CTCTGAACAGGAAACACGGT
LED-23	CAGGTCAAGTCGGTTCTCAG
LED-24	CGCCAGACTCTAAAAAGGAA
LED-25	CCCTCAGGATTCTACTGACT
LED-26	CTGTGTCGAGTCTGTTTCAG
LED-27	GAGAGGAGTCTCAGTGCTAC
LED-28	GATTCTGCAGTAACCTTGCT
LED-29	GCCAGGTCCTTTATCTTCTC
LED-30	TCTGATGGGACAGAATTTGC
LED-31	CTCCTTGTCAGGATGCTCTG
LED-32	CATTAGCACCAGTGCGTTTG
LED-33	CAAGGACTGGGGTCCATTTC
LED-34	GGGGATAAAGAGGACCATCT
LED-35	ATCTTGCTTTGCCAAGTCAT
LED-36	TTTTCCTCTGTAGCAGGGAT
LED-37	TAAAAACTCCAGGTTGTGCA
LED-38	CGTATTCACTGCTTACAAGC
LED-39	TTCTTCTTGGGGCTCTGTTT
LED-40	CACTGGTAGCTCAGGAAAAG
LED-41	GGTCATCTTCCATCTTTGCT
LED-42	GGATTTCTTCTGGACCTCAC
LED-43	CATTTGGAGAAGGCTCTCAG
LED-44	AAGACAGGTAGACCCATCTC
LED-45	GAAACTTCCCACTTAGCTGC
LED-46	ATGTATTTGCTCTAGTGCCC
LED-47	GGTGCACACAAAGTGAAAAT
LED-48	GCGGAATCAGATTTCAAAGT
**Chirp**	**modified with C3(biotin)**
LacZ_1	CCAGTGAATCCGTAATCATG
LacZ_2	TCACGACGTTGTAAAACGAC
LacZ_3	ATTAAGTTGGGTAACGCCAG
LacZ_4	AGGTTACGTTGGTGTAGATG
LacZ_5	AATGTGAGCGAGTAACAACC
LacZ_6	GTAGCCAGCTTTCATCAACA
LacZ_7	AATAATTCGCGTCTGGCCTT
LacZ_8	AGATGAAACGCCGAGTTAAC
LacZ_9	AATTCAGACGGCAAACGACT
LacZ_10	TTTCTCCGGCGCGTAAAAAT
LacZ_11	ATCTTCCAGATAACTGCCGT
LacZ_12	AACGAGACGTCACGGAAAAT

LacZ_13	GCTGATTTGTGTAGTCGGTT
LacZ_14	TTAAAGCGAGTGGCAACATG
LacZ_15	AACTGTTACCCGTAGGTAGT
LacZ_16	ATAATTTCACCGCCGAAAGG
LacZ_17	TTTCGACGTTCAGACGTAGT
LacZ_18	ATAGAGATTCGGGATTTCGG
LacZ_19	ACCATTTTCAATCCGCACCT
LacZ_20	TTAACGCCTCGAATCAGCAA
LacZ_21	TTCATCAGCAGGATATCCTG
LacZ_22	CACGGCGTTAAAGTTGTTCT
LacZ_23	TGGTTCGGATAATGCGAACA
LacZ_24	TTGGCTTCATCCACCACATA
LacZ_25	AGACGATTCATTGGCACCAT
LacZ_26	TGATCACACTCGGGTGATTA
LacZ_27	ATTTGATCCAGCGATACAGC
LacZ_28	AAATAATATCGGTGGCCGTG
LacZ_29	TTTGATGGACCATTTCGGCA
LacZ_30	ATTTAGCGAAACCGCCAAGA
LacZ_31	AAACGGGGATACTGACGAAA
LacZ_32	TTAATCAGCGACTGATCCAC
LacZ_33	ATACAGAACTGGCGATCGTT
LacZ_34	AAACTGCTGCTGGTGTTTTG
LacZ_35	TATTCGCTGGTCACTTCGAT
LacZ_36	GTTATCGCTATGACGGAACA
LacZ_37	TTTACCTTGTGGAGCGACAT
LacZ_38	GTTCAGGCAGTTCAATCAAC
LacZ_39	AAATCCATTTCGCTGGTGGT
LacZ_40	TTGCCAACGCTTATTACCCA
LacZ_41	TGTGAAAGAAAGCCTGACTG
LacZ_42	GGCGTCAGCAGTTGTTTTTT
LacZ_43	TACGCCAATGTCGTTATCCA
LacZ_44	TAAGGTTTTCCCCTGATGCT
LacZ_45	ATCAATCCGGTAGGTTTTCC
LacZ_46	GTAATCGCCATTTGACCACT
LacZ_47	AGTTTTCTTGCGGCCCTAAT
LacZ_48	ATGTCTGACAATGGCAGATC
**RNA FISH**	**modified with Quasar 570**
LED-1	GTAGTTTCTGGAGCCCGATG
LED-2	CCCTCTTTGGCCAATATGAG
LED-3	CAAATGCCAATGCCTTTCTG
LED-4	GGGAACTCAGACCTTCCTTC
LED-5	CAAGGAAAGCGACATTCCTA
LED-6	GATCTTCCCTGTGACTTTCC
LED-7	AGCATTGTACACCAGTTAGC
LED-8	GTTTTCAGTCCCAGGAACTG
LED-9	CAGCTTCATTCAGGACCTGA
LED-10	TGTCACTCTTGTCCCATAGA

LED-11	ATCTGTGCATCAGACACAAG
LED-12	TGACACCAGGGTTTGAAAGG
LED-13	CTCAGCAAAGAGCTTTCCTC
LED-14	CGCAACTTTATCCCAGTAGG
LED-15	GACTTCCGTGACCCTCTTAT
LED-16	ACCGCCTAGAAAACAGACTG
LED-17	GTATGTCATTCCGGTCAGTG
LED-18	ACACAGTGAATCACACACAG
LED-19	AAACAAGAACAGCTCTCTGG
LED-20	ACAAGAAGACGTGACAACAC
LED-21	GCCTCCAGTTGGGATTTTAG
LED-22	CTCTGAACAGGAAACACGGT
LED-23	CAGGTCAAGTCGGTTCTCAG
LED-24	CGCCAGACTCTAAAAAGGAA
LED-25	CCCTCAGGATTCTACTGACT
LED-26	CTGTGTCGAGTCTGTTTCAG
LED-27	GAGAGGAGTCTCAGTGCTAC
LED-28	GATTCTGCAGTAACCTTGCT
LED-29	GCCAGGTCCTTTATCTTCTC
LED-30	TCTGATGGGACAGAATTTGC
LED-31	CTCCTTGTCAGGATGCTCTG
LED-32	CATTAGCACCAGTGCGTTTG
LED-33	CAAGGACTGGGGTCCATTTC
LED-34	GGGGATAAAGAGGACCATCT
LED-35	ATCTTGCTTTGCCAAGTCAT
LED-36	TTTTCCTCTGTAGCAGGGAT
LED-37	TAAAAACTCCAGGTTGTGCA
LED-38	CGTATTCACTGCTTACAAGC
LED-39	TTCTTCTTGGGGCTCTGTTT
LED-40	CACTGGTAGCTCAGGAAAAG
LED-41	GGTCATCTTCCATCTTTGCT
LED-42	GGATTTCTTCTGGACCTCAC
LED-43	CATTTGGAGAAGGCTCTCAG
LED-44	AAGACAGGTAGACCCATCTC
LED-45	GAAACTTCCCACTTAGCTGC
LED-46	ATGTATTTGCTCTAGTGCCC
LED-47	GGTGCACACAAAGTGAAAAT
LED-48	GCGGAATCAGATTTCAAAGT
**Northern probes**	**modified with TEG-biotin**
LED	CTTTATTTATTTGCAGTCTGTTTTCTAGGC
p21e	CCTTTGGCCTTTTTAGCTGTATGACTCAGG

Supplementary Table 1. List of oligonucleotide sequences

SUPPLEMENTARY TABLE 2

chromosome	start	end
chr2	20451349	20451477
chr2	20453583	20453775
chr2	20454624	20454745
chr2	20454977	20455102
chr2	20455794	20455931
chr2	20457998	20458132
chr2	20460098	20460227
chr2	20462954	20463221
chr2	20478344	20478580
chr2	20482708	20482992
chr2	20483104	20483247
chr2	20490413	20490551
chr2	20494137	20494279
chr2	20497314	20497439
chr2	20507739	20507832
chr2	20508075	20508345
chr2	20511255	20511424
chr2	20511997	20512184
chr2	20518298	20518406
chr2	20526093	20526146
chr2	20527071	20527121
chr3	156864345	156864396
chr3	156866033	156866378
chr3	156867076	156867174
chr3	156867274	156867385
chr3	156867602	156867766
chr3	156867854	156867958
chr3	156868071	156868170
chr3	156869698	156869719
chr3	156869966	156870030
chr3	156870825	156870945
chr3	156874845	156874872
chr3	156876655	156876764
chr3	156877194	156877268
chr3	156877581	156877883
chr5	126113201	126113559
chr5	126140468	126140624
chr5	126141263	126141388
chr5	126145872	126146042
chr5	126147465	126147590
chr5	126154614	126154835
chr5	126156602	126156827

chr5	126158473	126158577
chr5	126161680	126161799
chr5	126168386	126168493
chr5	126171915	126171953
chr14	21966405	21966513
chr14	21967169	21967281
chr14	21967450	21967515
chr14	21967636	21967744
chr14	21967908	21967946
chr14	21968637	21968824
chr14	21969055	21969271
chr14	21969870	21970045
chr14	21971316	21971720
chr14	21971807	21972024
chr14	21979266	21979365
chr15	66161924	66161963
chr15	66169670	66169865
chr15	66170100	66170293
chr15	66170369	66170377
chr15	66172009	66172089
chr15	66180039	66180175
chr16	53738097	53738141
chr16	53844052	53844129
chr16	53859776	53860403
chr16	53878067	53878210
chr16	53907698	53907777
chr16	53913756	53913899
chr16	53922744	53922863
chr16	53945292	53945396
chr16	53967897	53968021
chr16	54018850	54018866
chr16	54145674	54145824
chr16	70328749	70328820
chr16	70333201	70333257
chr16	70346512	70346560
chr16	70348805	70348858
chr16	70349872	70350007
chr16	70351399	70351491
chr16	70358488	70358590
chr16	70359477	70359591
chr16	70363195	70363372
chr16	70363734	70363971
chr16	70365625	70365787
chr16	70366871	70367062
chr16	70367424	70367482

chr19	48248817	48249040
chr19	48250224	48250288
chr19	48253435	48253543
chr19	48254165	48254364
chr19	48254777	48254847
chr19	48255769	48255864
chr19	48257784	48257888
chr19	48257966	48258148
chr19	48258605	48258780
chr19	48259013	48259079
chr19	48259785	48259861
chr19	48259945	48260001
chr19	48260256	48260259
chr20	32264541	32264785
chr20	32264911	32265136
chr20	32265232	32265346
chr20	32266007	32266159
chr20	32267561	32267780
chr20	32268132	32268222
chr20	32273810	32274070
chrX	134229015	134229254
chrX	134229530	134229594
chrX	134230877	134231151
chrX	134232063	134232654
chrX	134555868	134556655
chrX	134557558	134559682

Supplementary Table 2. ChIRP peak list of plasmid contaminants
